# Supplementary material for: Circulation of a digital community currency
Source: Sci Rep. 2023 Apr 11;13:5864. doi: 10.1038/s41598-023-33184-1 (PMC10088680; doi:10.1038/s41598-023-33184-1)
Supplement: Supplementary file 1 — Supplementary Information 1. [file 41598_2023_33184_MOESM1_ESM.html]

SI\_1


In [1]:

```
import numpy as np
import pandas as pd
import json
import re
import os
import math
import random 
%matplotlib inline
```

In [2]:

```
np.__version__
```

Out[2]:

```
'1.22.3'
```

In [3]:

```
pd.__version__
```

Out[3]:

```
'1.4.2'
```

## Directory paths & transaction data¶

In [4]:

```
# Define directories
homedir = os.path.expanduser("~")
projdir = os.path.join(homedir,'Documents','Research','Sarafu')
datadir = os.path.join(projdir,'Sarafu2021_UKDS')
figsdur = os.path.join(projdir,'Exploration','figures')
```

In [5]:

```
# Load into pandas
raw_fn = os.path.join(datadir,"sarafu_xdai","sarafu_txns_20200125-20210615.csv")
raw = pd.read_csv(raw_fn).drop(columns=['token_name','token_address'])
```

In [6]:

```
# User data
users_fn = os.path.join(datadir,"sarafu_xdai","sarafu_users_20210615.csv")
categoricals = ['gender','area_name','area_type','held_roles','business_type']
strings = ['start','old_POA_blockchain_address','xDAI_blockchain_address']
dtypes = {col:"category" for col in categoricals}
dtypes.update({col:"string" for col in strings})
users = pd.read_csv(users_fn,dtype=dtypes,na_filter=False)
users = users.drop_duplicates(subset=['xDAI_blockchain_address'],keep='first')
users = users.set_index('xDAI_blockchain_address')
```

## Data cleaning¶

Removing transactions directly between system-run accounts to avoid them appearing as user accounts.

In [7]:

```
# These are few & small in size
admins = ['0xBDB3Bc887C3b70586BC25D04d89eC802b897fC5F','0xEDA5C9B75Fdb3B9bdAB987A704632280Cf93084F']
raw[(raw['source'].isin(admins)) & (raw['target'].isin(admins))]
```

Out[7]:

|  | id | timeset | transfer\_subtype | source | target | weight |
| --- | --- | --- | --- | --- | --- | --- |
| 98019 | 148297 | 2020-04-27 18:40:43.397483 | RECLAMATION | 0xBDB3Bc887C3b70586BC25D04d89eC802b897fC5F | 0xEDA5C9B75Fdb3B9bdAB987A704632280Cf93084F | 20.0 |
| 100138 | 150433 | 2020-04-27 19:18:51.213185 | RECLAMATION | 0xBDB3Bc887C3b70586BC25D04d89eC802b897fC5F | 0xEDA5C9B75Fdb3B9bdAB987A704632280Cf93084F | 20.0 |
| 101632 | 151933 | 2020-04-27 19:45:03.398842 | RECLAMATION | 0xBDB3Bc887C3b70586BC25D04d89eC802b897fC5F | 0xEDA5C9B75Fdb3B9bdAB987A704632280Cf93084F | 20.0 |
| 103317 | 153621 | 2020-04-27 20:16:11.444973 | RECLAMATION | 0xBDB3Bc887C3b70586BC25D04d89eC802b897fC5F | 0xEDA5C9B75Fdb3B9bdAB987A704632280Cf93084F | 20.0 |
| 103326 | 153630 | 2020-04-27 20:16:25.915760 | RECLAMATION | 0xBDB3Bc887C3b70586BC25D04d89eC802b897fC5F | 0xEDA5C9B75Fdb3B9bdAB987A704632280Cf93084F | 20.0 |
| 103389 | 153693 | 2020-04-27 20:18:19.326710 | RECLAMATION | 0xBDB3Bc887C3b70586BC25D04d89eC802b897fC5F | 0xEDA5C9B75Fdb3B9bdAB987A704632280Cf93084F | 20.0 |
| 103390 | 153694 | 2020-04-27 20:18:20.821020 | RECLAMATION | 0xBDB3Bc887C3b70586BC25D04d89eC802b897fC5F | 0xEDA5C9B75Fdb3B9bdAB987A704632280Cf93084F | 20.0 |
| 103391 | 153695 | 2020-04-27 20:18:22.363205 | RECLAMATION | 0xBDB3Bc887C3b70586BC25D04d89eC802b897fC5F | 0xEDA5C9B75Fdb3B9bdAB987A704632280Cf93084F | 20.0 |
| 103392 | 153696 | 2020-04-27 20:18:23.901366 | RECLAMATION | 0xBDB3Bc887C3b70586BC25D04d89eC802b897fC5F | 0xEDA5C9B75Fdb3B9bdAB987A704632280Cf93084F | 20.0 |
| 103393 | 153697 | 2020-04-27 20:18:25.426123 | RECLAMATION | 0xBDB3Bc887C3b70586BC25D04d89eC802b897fC5F | 0xEDA5C9B75Fdb3B9bdAB987A704632280Cf93084F | 20.0 |
| 103394 | 153698 | 2020-04-27 20:18:26.952261 | RECLAMATION | 0xBDB3Bc887C3b70586BC25D04d89eC802b897fC5F | 0xEDA5C9B75Fdb3B9bdAB987A704632280Cf93084F | 20.0 |
| 103398 | 153702 | 2020-04-27 20:18:34.094907 | RECLAMATION | 0xBDB3Bc887C3b70586BC25D04d89eC802b897fC5F | 0xEDA5C9B75Fdb3B9bdAB987A704632280Cf93084F | 20.0 |
| 103399 | 153703 | 2020-04-27 20:18:35.584158 | RECLAMATION | 0xBDB3Bc887C3b70586BC25D04d89eC802b897fC5F | 0xEDA5C9B75Fdb3B9bdAB987A704632280Cf93084F | 20.0 |
| 339084 | 434034 | 2020-08-05 14:54:57.588080 | DISBURSEMENT | 0xBDB3Bc887C3b70586BC25D04d89eC802b897fC5F | 0xBDB3Bc887C3b70586BC25D04d89eC802b897fC5F | 1.0 |
| 352684 | 447899 | 2020-08-11 14:58:38.895721 | DISBURSEMENT | 0xBDB3Bc887C3b70586BC25D04d89eC802b897fC5F | 0xBDB3Bc887C3b70586BC25D04d89eC802b897fC5F | 1.0 |

In [8]:

```
# Note the filtered transactions
remv_fn = os.path.join(datadir,"transactions","sarafu_txns_removed.csv")
remv = raw[(raw['source'].isin(admins)) & (raw['target'].isin(admins))]
remv.to_csv(remv_fn,index=False)
```

In [9]:

```
# Save the resulting dataset
txns_fn = os.path.join(datadir,"transactions","sarafu_txns.csv")
txns = raw[~((raw['source'].isin(admins)) & (raw['target'].isin(admins)))].copy()
txns.to_csv(txns_fn,index=False)
```

## STANDARD transactions¶

In [10]:

```
# Filter out the currency management and cash exchange operations
txns_std = txns[txns['transfer_subtype']=="STANDARD"].copy()
```

##### Timestamps¶

In [11]:

```
from pandas.api.types import CategoricalDtype
from datetime import datetime, timedelta
```

In [12]:

```
# generate timestamp
txns_std['timestamp'] = pd.to_datetime(txns_std['timeset'],format="%Y-%m-%d %H:%M:%S.%f")
```

In [13]:

```
# month
txns_std['Month'] = txns_std['timestamp'].dt.strftime("%b %y")
category = CategoricalDtype(categories=reversed(['Jan 20','Feb 20','Mar 20','Apr 20','May 20','Jun 20',
                                        'Jul 20','Aug 20','Sep 20','Oct 20','Nov 20','Dec 20',
                                        'Jan 21','Feb 21','Mar 21','Apr 21','May 21','Jun 21']),ordered=True)
txns_std['Month'] = txns_std['Month'].astype(category)
```

##### Timeseries of STANDARD transactions¶

In [14]:

```
# Give each transaction the greographic area of the source_ID
mapping = users['area_name'].to_dict()
txns_std["source_area"] = txns_std["source"].apply(lambda x: mapping[x])
category = CategoricalDtype(categories=['Mukuru Nairobi','Misc Nairobi',
                                        'Kinango Kwale','Kilifi',
                                        'Kisauni Mombasa','Misc Mombasa',
                                        'Nyanza','Turkana',
                                        'Misc Rural Counties','other'],ordered=True)
txns_std["source_area"] = txns_std['source_area'].astype(category)
txns_std["source_area"] = txns_std['source_area'].cat.rename_categories({'Misc Rural Counties': 'Misc Rural'})
```

In [15]:

```
# Monthly volumes of STANDARD transactions -- February through May
feb20 = datetime.strptime("2020-02-01 00:00:00.00","%Y-%m-%d %H:%M:%S.%f")
jun21 = datetime.strptime("2021-06-01 00:00:00.00","%Y-%m-%d %H:%M:%S.%f")

# filter out incomplete months 
txns_febmay = txns_std[(txns_std['timestamp']>feb20)&(txns_std['timestamp']<jun21)].copy()
txns_febmay["Month"] = txns_febmay["Month"].cat.remove_unused_categories()
# compute totals
monthly = txns_febmay[["Month","weight"]].groupby(['Month']).sum()
# compute totals per geographic area
monthlies = txns_febmay[["Month","source_area","weight"]].groupby(['Month',"source_area"]).sum()
# reconstitute timestamps
monthly = monthly.reset_index()
monthlies = monthlies.reset_index()
monthly["month"] = pd.to_datetime(monthly["Month"],format="%b %y")+timedelta(days=15)
monthlies["month"] = pd.to_datetime(monthlies["Month"],format="%b %y").astype("datetime64[ns]")+timedelta(days=15)
```

##### Plot¶

In [16]:

```
import matplotlib as mpl
import matplotlib.pyplot as plt
import matplotlib.dates as mdates
import matplotlib.ticker as ticker
import seaborn as sns
```

In [17]:

```
mpl.__version__
```

Out[17]:

```
'3.5.2'
```

In [18]:

```
sns.__version__
```

Out[18]:

```
'0.11.2'
```

In [19]:

```
# Gephi-matching custom colormap
color = {'Mukuru Nairobi':"#85BD39",
         'Misc Nairobi':"#2A6425",
         'Kinango Kwale':"#D390F8",
         'Kilifi':"#303030",
         'Kisauni Mombasa':"#54C0F8",
         'Misc Mombasa':"#0425BF",
         'Nyanza':"#54BBAA",
         'Turkana':"#F28D25",
         'Misc Rural':"#A03918",
         'other':"#A2A2A2"
        }
```

In [20]:

```
# generate figure
filepath = os.path.join(figsdur,"volume_monthly.pdf")
fig, ax = plt.subplots(figsize=(6.5,4))
sns.lineplot(data=monthly,
            x="month",
            y="weight",
            color="black",
            linewidth=2,
            label="Total",
            ax=ax)
sns.lineplot(data=monthlies,
            x="month",
            y="weight",
            hue="source_area",
            palette=sns.color_palette([color[area] for area in txns_std["source_area"].cat.categories]),
            linewidth=1,
            ax=ax)

# Bump legend right 
sns.move_legend(ax, title = None, loc = 2, bbox_to_anchor = (1,1), frameon=False)
# Axis labels
ax.set_ylabel("Transaction volume (Sarafu)",fontsize=12.0)
ax.set_xlim(feb20,jun21-timedelta(days=1))
locator = mdates.MonthLocator()  # every month
# Specify the format - %b gives us Jan, Feb...
fmt = mdates.DateFormatter('%b %y')
X = plt.gca().xaxis
X.set_major_locator(locator)
# Specify formatter
X.set_major_formatter(fmt)
ax.tick_params(axis='x', labelrotation=45)
ax.set_xlabel(None)
# Hide the right and top spines
ax.spines['right'].set_visible(False)
ax.spines['top'].set_visible(False)
# Bump legend right

plt.tight_layout()
plt.savefig(filepath)
plt.show()
```

In [21]:

```
# generate figure
filepath = os.path.join(figsdur,"volume_monthly_detail.pdf")
fig, ax = plt.subplots(figsize=(5,4))
sns.lineplot(data=monthly,
            x="month",
            y="weight",
            color="black",
            linewidth=2,
            label="Total",
            ax=ax)
sns.lineplot(data=monthlies,
            x="month",
            y="weight",
            hue="source_area",
            palette=sns.color_palette([color[area] for area in txns_std["source_area"].cat.categories]),
            linewidth=1,
            ax=ax)
# Axis labels
ax.set_ylabel("Transaction volume (Sarafu)",fontsize=12.0)
ax.set_xlim(feb20,jun21-timedelta(days=1))
# Specify the format - %b gives us Jan, Feb...
locator = mdates.MonthLocator()  # every month
ax.xaxis.set_major_locator(locator)
# Specify formatter
fmt = mdates.DateFormatter('%b %y')
ax.xaxis.set_major_formatter(fmt)
ax.tick_params(axis='x', labelrotation=45, labelsize=11.0)
ax.set_xlabel(None)
# Hide the right and top spines
ax.spines['right'].set_visible(False)
ax.spines['top'].set_visible(False)
# Bump legend right 
tmp = ax.legend(bbox_to_anchor=(1.65,1), title = None, loc = 2, frameon=False, fontsize=11.0)

# Insert
ax2 = plt.axes([0.8, 0.42, .6, .45])

sns.lineplot(data=monthlies[~monthlies['source_area'].isin(["Mukuru Nairobi","Misc Nairobi","Kinango Kwale"])],
            x="month",
            y="weight",
            hue="source_area",
            palette=sns.color_palette([color[area] for area in txns_std["source_area"].cat.categories]),
            linewidth=1,
            legend=False,
            ax=ax2)

# Axis labels
ax2.set_ylabel(None)
ax2.ticklabel_format(style='sci', axis='y',scilimits=(0,1))
ax2.set_xlim(feb20,jun21-timedelta(days=1))
# Specify the format - %b gives us Jan, Feb...
locator = mdates.MonthLocator()  # every month
ax2.xaxis.set_major_locator(locator)
# Specify formatter
fmt = mdates.DateFormatter('%b %y')
ax2.xaxis.set_major_formatter(fmt)
ax2.tick_params(axis='x', labelrotation=45, labelsize=9.0)
ax2.set_xlabel(None)
# Hide the right and top spines
ax2.spines['right'].set_visible(False)
ax2.spines['top'].set_visible(False)

plt.savefig(filepath,bbox_extra_artists=(tmp,), bbox_inches='tight')
plt.show()
```

## Sarafu flow network¶

In [22]:

```
# Total Sarafu over each link
flow_net = txns_std.groupby(by = ['source', 'target']).sum().reset_index().drop(columns=['id'])
```

In [23]:

```
# Print the flow network
os.makedirs(os.path.join(datadir,"networks"), exist_ok=True)
flow_net_fn = os.path.join(datadir,"networks","sarafu_flow_network.csv")
flow_net.to_csv(flow_net_fn,index=False)
```

### Networkx¶

In [24]:

```
import networkx as nx
nx.__version__
```

Out[24]:

```
'2.6.3'
```

In [25]:

```
# Edgelist
flow_nx = nx.from_pandas_edgelist(flow_net,edge_attr='weight',create_using=nx.DiGraph)
```

In [26]:

```
# Node information
nx.set_node_attributes(flow_nx, users.to_dict('index'))
```

In [27]:

```
print("nodes", flow_nx.number_of_nodes())
print("edges", flow_nx.number_of_edges())
```

```
nodes 40767
edges 146615
```

##### Filter & format¶

In [28]:

```
# Filter system accounts
has_admin_role = users['held_roles'].isin(['ADMIN','VENDOR'])
has_admin_type = users['business_type'].isin(['system'])
reg_users = users.loc[~has_admin_role & ~has_admin_type].copy()
```

In [29]:

```
# Gather the network subgraph
flow_reg_nx = nx.DiGraph()
for e, e_dict in flow_nx.subgraph(reg_users.index).edges.items():
    flow_reg_nx.add_edge(*e,**e_dict)
```

In [30]:

```
# write in Pajek format
flow_reg_fn = os.path.join(datadir,"networks","sarafu_reg_users.net")
nx.write_pajek(flow_reg_nx, flow_reg_fn, encoding='UTF-8')
# clean up the file
with open(flow_reg_fn, 'r') as file :
    filedata = file.read()
filedata = filedata.replace(' 0.0 0.0 ellipse', '')
with open(flow_reg_fn, 'w') as file:
    file.write(filedata)
```

In [31]:

```
print(" nodes", flow_reg_nx.number_of_nodes())
print(" edges", flow_reg_nx.number_of_edges())
print("weight", flow_reg_nx.size(weight="weight"))
```

```
 nodes 40657
 edges 145661
weight 293688301.0480015
```

In [32]:

```
flow_reg_gcc_nx = flow_reg_nx.subgraph(max(nx.weakly_connected_components(flow_reg_nx), key=len))
print(" nodes gcc", flow_reg_gcc_nx.number_of_nodes())
print(" edges gcc", flow_reg_gcc_nx.number_of_edges())
print("weight gcc", flow_reg_gcc_nx.size(weight="weight"))
```

```
 nodes gcc 38653
 edges gcc 143724
weight gcc 293378855.0480015
```

In [33]:

```
# Again with counts, to get the number of transactions this represents
txns_net = txns_std.groupby(by = ['source', 'target']).count().reset_index().drop(columns=['id'])
txns_nx = nx.from_pandas_edgelist(txns_net,edge_attr='weight',create_using=nx.DiGraph)
nx.set_node_attributes(txns_nx, users.to_dict('index'))
txns_reg_nx = nx.DiGraph()
for e, e_dict in txns_nx.subgraph(reg_users.index).edges.items():
    txns_reg_nx.add_edge(*e,**e_dict)
```

In [34]:

```
print(" nodes", txns_reg_nx.number_of_nodes())
print(" edges", txns_reg_nx.number_of_edges())
print("weight", txns_reg_nx.size(weight="weight"))
```

```
 nodes 40657
 edges 145661
weight 421329.0
```

In [35]:

```
txns_reg_gcc_nx = txns_reg_nx.subgraph(max(nx.weakly_connected_components(txns_reg_nx), key=len))
print(" nodes gcc", txns_reg_gcc_nx.number_of_nodes())
print(" edges gcc", txns_reg_gcc_nx.number_of_edges())
print("weight gcc", txns_reg_gcc_nx.size(weight="weight"))
```

```
 nodes gcc 38653
 edges gcc 143724
weight gcc 418675.0
```

#### Non-STANDARD¶

In [36]:

```
reg_nodes = set(flow_reg_nx.nodes())
```

In [37]:

```
# Filter in the currency management and cash exchange operations
txns_nonstd = txns[txns['transfer_subtype']!="STANDARD"].copy()
```

In [38]:

```
# Make a network
flow_inout = txns_nonstd.groupby(by = ['source', 'target']).sum().reset_index().drop(columns=['id'])
flow_inout_nx = nx.from_pandas_edgelist(flow_inout,edge_attr='weight',create_using=nx.DiGraph)
```

In [39]:

```
# collect non-STANDARD in/out-flows
nx.set_node_attributes(flow_reg_nx, 0, "nonstd_in")
nx.set_node_attributes(flow_reg_nx, 0, "nonstd_out")
nx.set_node_attributes(flow_reg_nx, {node:val for node, val in flow_inout_nx.in_degree(weight='weight') if node in reg_nodes}, "nonstd_in")
nx.set_node_attributes(flow_reg_nx, {node:val for node, val in flow_inout_nx.out_degree(weight='weight') if node in reg_nodes}, "nonstd_out")
```

In [40]:

```
# make a dataframe
flow_reg_nodes = pd.DataFrame.from_dict(dict(flow_reg_nx.nodes(data=True)),orient='index')
flow_reg_nodes = flow_reg_nodes.join(reg_users[["ovol_in"]])
```

In [41]:

```
# correlations
print('nonstandard',np.corrcoef(flow_reg_nodes["ovol_in"],flow_reg_nodes['nonstd_in'])[1,0])
```

```
nonstandard 0.9999999947281789
```

In [42]:

```
# not enough to worry about; we use the pre-computed ovol_in value already in the user dataset
```

##### Transactions with system-run accounts¶

In [43]:

```
# total STANDARD
nx.set_node_attributes(flow_reg_nx, {node:val for node, val in flow_nx.in_degree(weight='weight') if node in reg_nodes}, "std_in")
nx.set_node_attributes(flow_reg_nx, {node:val for node, val in flow_nx.out_degree(weight='weight') if node in reg_nodes}, "std_out")
# total regular
nx.set_node_attributes(flow_reg_nx, {node:val for node, val in flow_reg_nx.in_degree(weight='weight') if node in reg_nodes}, "reg_in")
nx.set_node_attributes(flow_reg_nx, {node:val for node, val in flow_reg_nx.out_degree(weight='weight') if node in reg_nodes}, "reg_out")
```

In [44]:

```
# now a dataframe
flow_reg_nodes = pd.DataFrame.from_dict(dict(flow_reg_nx.nodes(data=True)),orient='index')
flow_reg_nodes = flow_reg_nodes.join(reg_users[["ovol_in"]])
# total
flow_reg_nodes['tot_in'] = flow_reg_nodes['nonstd_in'] + flow_reg_nodes['std_in']
flow_reg_nodes['tot_out'] = flow_reg_nodes['nonstd_out'] + flow_reg_nodes['std_out']
# total irregular
flow_reg_nodes['irr_in'] = flow_reg_nodes['tot_in'] - flow_reg_nodes['reg_in']
flow_reg_nodes['irr_out'] = flow_reg_nodes['tot_out'] - flow_reg_nodes['reg_out']
```

In [45]:

```
# correlations
print(' irregular',np.corrcoef(flow_reg_nodes["ovol_in"],flow_reg_nodes['irr_in'])[1,0])
```

```
 irregular 0.9823694550189346
```

In [46]:

```
# not enough to worry about; better to use just the non-STANDARD inflows, i.e. the pre-computed ovol_in value
# the same accounts with inflow from system accounts likely also have outflows, which anyways isn't modelled
```

In [ ]:

```
! jupyter nbconvert --to html your_notebook_name.ipynb
```
