# Supplementary material for: Circulation of a digital community currency
Source: Sci Rep. 2023 Apr 11;13:5864. doi: 10.1038/s41598-023-33184-1 (PMC10088680; doi:10.1038/s41598-023-33184-1)
Supplement: Supplementary file 2 — Supplementary Information 2. [file 41598_2023_33184_MOESM2_ESM.html]

SI\_2


In [1]:

```
import numpy as np
import pandas as pd
import json
import re
import os
import math
import random 
from collections import Counter
from datetime import datetime, timedelta
import networkx as nx
import matplotlib.pyplot as plt
import matplotlib.dates as mdates
import seaborn as sns
%matplotlib inline
```

## Directory paths & data¶

In [2]:

```
# Define directories
homedir = os.path.expanduser("~")
datadir = os.path.join(homedir,'Documents','Research','Sarafu','Sarafu2021_UKDS')
projdir = os.path.join(homedir,'Documents','Research','Sarafu','Exploration')

os.makedirs(os.path.join(projdir,"figures"), exist_ok=True)
os.makedirs(os.path.join(projdir,"analysis"), exist_ok=True)

figsdir = os.path.join(projdir,'figures')
```

In [3]:

```
# User data
users_fn = os.path.join(datadir,"sarafu_xdai","sarafu_users_20210615.csv")
categoricals = ['gender','area_name','area_type','held_roles','business_type']
strings = ['start','old_POA_blockchain_address','xDAI_blockchain_address']
dtypes = {col:"category" for col in categoricals}
dtypes.update({col:"string" for col in strings})
users = pd.read_csv(users_fn,dtype=dtypes,na_filter=False)
users = users.drop_duplicates(subset=['xDAI_blockchain_address'],keep='first')
users = users.set_index('xDAI_blockchain_address')
```

##### Overall and cumulative flow networks¶

In [4]:

```
from datetime import datetime, timedelta
import networkx as nx
```

In [5]:

```
# Flow network
flow_reg_fn = os.path.join(datadir,"networks","sarafu_reg_users.net")
flow_reg_nx = nx.DiGraph(nx.read_pajek(flow_reg_fn,encoding='UTF-8'))
```

In [6]:

```
# Grab the number of nodes & total volume
print("nodes",flow_reg_nx.number_of_nodes())
print("edges",flow_reg_nx.size())
print("volume",flow_reg_nx.size(weight="weight"))
```

```
nodes 40657
edges 145661
volume 293688301.0480015
```

##### Derived user data¶

In [7]:

```
# 'start' is a reserved keyword in Gephi
users['registered'] = users['start'] 
users = users.drop(['start'],axis=1)
```

In [8]:

```
# Year, month, day registered
users['reg_day'] = users['registered'].apply(lambda x: x.split(" ")[0])
users['reg_month'] = users['reg_day'].apply(lambda x: "-".join(x.split("-")[:2]))
users['reg_year'] = users['reg_day'].apply(lambda x: "-".join(x.split("-")[:1]))
```

In [9]:

```
# Days since registration
day_data = datetime.strptime('2021-06-15 00:00:00','%Y-%m-%d %H:%M:%S')

def days_old(registered):
    try:
        registered = datetime.strptime(registered,'%Y-%m-%d %H:%M:%S.%f')
    except:
        registered = datetime.strptime(registered,'%Y-%m-%d %H:%M:%S')
    return (day_data-registered).days

users['reg_age'] = users['registered'].apply(lambda x: days_old(x))
```

In [10]:

```
# Prior currency
users['prior'] = ~users['old_POA_blockchain_address'].apply(lambda x: x=="")
```

In [11]:

```
# Filter system accounts
has_admin_role = users['held_roles'].isin(['ADMIN','VENDOR'])
has_admin_type = users['business_type'].isin(['system'])
reg_users = users.loc[~has_admin_role & ~has_admin_type]
```

##### Node attributes¶

In [12]:

```
# Flow network
nx.set_node_attributes(flow_reg_nx, reg_users.to_dict('index'))
```

## Infomap¶

In [13]:

```
# create an 'infomap' folder within the 'analysis' directory, and
# run Infomap using the following script, or via the command line:
```

```
#!/bin/bash  

DATA='/Users/mattssonc/Documents/Research/Sarafu/Sarafu2021_UKDS'
WORKING='/Users/mattssonc/Documents/Research/Sarafu/Exploration'

infomap --flow-model rawdir --tree $DATA/networks/sarafu_reg_users.net $WORKING/analysis/infomap/ > $WORKING/analysis/infomap/sarafu_reg_users.out
```

##### Load Infomap output¶

In [15]:

```
# Load measures & modules
reg_users_mod_fn = os.path.join(projdir,"analysis","infomap","sarafu_reg_users.tree")
reg_users_mod = pd.read_csv(reg_users_mod_fn,names=['module','flow','node','idx'],sep=" ",skiprows=8)
reg_users_mod['node'] = reg_users_mod['node'].apply(lambda x: x.strip('"'))
reg_users_mod['mod_1'] = reg_users_mod['module'].apply(lambda x: ':'.join(x.split(':')[:min(1,len(x))]))
reg_users_mod['mod_2'] = reg_users_mod['module'].apply(lambda x: ':'.join(x.split(':')[:min(2,len(x))]))
reg_users_mod['mod_3'] = reg_users_mod['module'].apply(lambda x: ':'.join(x.split(':')[:min(3,len(x))]))
reg_users_mod['mod_4'] = reg_users_mod['module'].apply(lambda x: ':'.join(x.split(':')[:min(4,len(x))]))
reg_users_mod['mod_5'] = reg_users_mod['module'].apply(lambda x: ':'.join(x.split(':')[:min(5,len(x))]))
reg_users_mod['mod_6'] = reg_users_mod['module'].apply(lambda x: ':'.join(x.split(':')[:min(6,len(x))]))
reg_users_mod['mod_7'] = reg_users_mod['module'].apply(lambda x: ':'.join(x.split(':')[:min(7,len(x))]))
reg_users_mod = reg_users_mod.drop(['idx','flow'],axis=1)
reg_users_mod = reg_users_mod.set_index('node')
```

In [16]:

```
# Set node attributes
reg_users = reg_users_mod.join(reg_users, how='left')
# Flow network
nx.set_node_attributes(flow_reg_nx, reg_users.to_dict('index'))
```

In [17]:

```
# Confirm consistent number of nodes
print("nodes",flow_reg_nx.number_of_nodes())
print("users",reg_users.index.size)
```

```
nodes 40657
users 40657
```

In [18]:

```
flow_reg_nx.nodes['0xfB9BC95038904F42654b6b8f0cf2c0F616C5261B']
```

Out[18]:

```
{'id': 18455,
 'final_bal': '77043.64',
 'gender': 'Female',
 'area_name': 'Mukuru Nairobi',
 'area_type': 'urban',
 'held_roles': 'GROUP_ACCOUNT',
 'business_type': 'savings',
 'old_POA_blockchain_address': '',
 'ovol_in': 3043.12,
 'ovol_out': 4834.48,
 'otxns_in': 19,
 'otxns_out': 7,
 'ounique_in': 3,
 'ounique_out': 1,
 'svol_in': 653405.0,
 'svol_out': 574570.0,
 'stxns_in': 133,
 'stxns_out': 90,
 'sunique_in': 57,
 'sunique_out': 46,
 'registered': '2020-05-01 10:25:54.211211',
 'reg_day': '2020-05-01',
 'reg_month': '2020-05',
 'reg_year': '2020',
 'reg_age': 409,
 'prior': False,
 'module': '1:3:1:4',
 'mod_1': '1',
 'mod_2': '1:3',
 'mod_3': '1:3:1',
 'mod_4': '1:3:1:4',
 'mod_5': '1:3:1:4',
 'mod_6': '1:3:1:4',
 'mod_7': '1:3:1:4'}
```

### Format & save¶

In [19]:

```
# Save as GEXF
flow_reg_gexf = os.path.join(datadir,"networks","sarafu_reg_users.gexf")
nx.write_gexf(flow_reg_nx,flow_reg_gexf)
```

In [20]:

```
#  # Our recipe for nicely visualizing the network in Gephi
#  - run directed, unweighted RageRank with alpha 0.85
#  - size nodes by PageRank, min 10 & max 50, with the third spline (a quarter circle)
#  - color nodes by area_name, match the colors to the legend of Figure 1
#  - filter to show only the giant component
#  - run "ForceAtlas 2" with default settings, except Gravity 80.0 and Edge Weight Influence 0.1
#      - this will take a while, and some nodes may get stuck so just help them along
#  - run "ForceAtlas 2" again, now with "Prevent Overlap" checked
#      - this takes maybe a minute
#  - center the network on the screen
#  < optional > 
#      - settle all the nodes in the giant component
#      - filter to show only NOT the giant component
#      - move the blob of small components out beyond the (hidden) giant component
#      - remove the filter
#      - run "ForceAtlas 2" with default settings, except Gravity 80.0 and Edge Weight Influence 0.1
#      - run "ForceAtlas 2" again, now with "Prevent Overlap" checked
#      - adjust as needed, toggling the filter on and off
#  - use "Expansion" twice at 1.2
#  - center the network on the screen
#  - output a png using the screenshot functionality
#      -  decent resolution: 2048x1536
#      -  medium resolution: 4096x3072
#      -  higher resolution: 8192x6144
#      - highest resolution: 16384x12288
```

##### Networks by top-level and second-level infomap module¶

In [21]:

```
# modules
mod_1s = ['1','2','3','4','5']
mod_2s = [key for key, value in Counter(dict(flow_reg_nx.nodes(data="mod_2")).values()).items() if value > 100]
mod_3s = [key for key, value in Counter(dict(flow_reg_nx.nodes(data="mod_3")).values()).items() if value > 10]
```

In [22]:

```
# Split the network by module
flow_reg_nxs = {}
print("layer","modules","in_weight","tot_weight","fraction")
for label, modules in [('mod_1',mod_1s),('mod_2',mod_2s),('mod_3',mod_3s)]:
    total_weight = 0
    flow_reg_nxs[label] = {}
    for module in modules:
        # Gather the network subgraph
        flow_reg_nxs[label][module] = nx.DiGraph()
        for e, e_dict in flow_reg_nx.subgraph(reg_users[reg_users[label]==module].index).edges.items():
            flow_reg_nxs[label][module].add_edge(*e,**e_dict)
        total_weight += flow_reg_nxs[label][module].size(weight="weight")
    print(label,len(modules),total_weight,flow_reg_nx.size(weight="weight"),total_weight/flow_reg_nx.size(weight="weight"))
```

```
layer modules in_weight tot_weight fraction
mod_1 5 292914508.23800033 293688301.0480015 0.9973652583121629
mod_2 37 283669368.11800003 293688301.0480015 0.9658858289749718
mod_3 455 235800600.764 293688301.0480015 0.8028940884691892
```

In [23]:

```
# write in Pajek format
for label, modules in [('mod_1',mod_1s),('mod_2',mod_2s)]:
    for module in modules:
        flow_reg_fn = os.path.join(datadir,"networks","modules",label,"sarafu_reg_users_"+module.replace(':',"~")+".net")
        nx.write_pajek(flow_reg_nxs[label][module], flow_reg_fn, encoding='UTF-8')
        # clean up the file
        with open(flow_reg_fn, 'r') as file :
            filedata = file.read()
        filedata = filedata.replace(' 0.0 0.0 ellipse', '')
        with open(flow_reg_fn, 'w') as file:
            file.write(filedata)
```

#### Geographic sorting¶

In [24]:

```
mod_1_users = reg_users[reg_users['mod_1'].isin(['1','2','3','4','5'])]
mod_1_pivot = pd.pivot_table(mod_1_users,values="id",index="area_name",columns='mod_1',aggfunc='count',fill_value=0)
mod_1_pivot = mod_1_pivot.reindex(['Kinango Kwale',
                                    'Mukuru Nairobi','Misc Nairobi',
                                    'Kilifi','Kisauni Mombasa','Misc Mombasa',
                                    'Nyanza','Turkana','other','Misc Rural Counties'])
mod_1_pivot = mod_1_pivot[['2','1','3','4','5']]
```

In [25]:

```
mod_2_users = reg_users[reg_users['mod_2'].isin(['3:1','3:2','3:3','3:4'])]
mod_2_pivot = pd.pivot_table(mod_2_users,values="id",index="area_name",columns='mod_2',aggfunc='count',fill_value=0)
mod_2_pivot = mod_2_pivot.reindex(['Kinango Kwale',
                                    'Mukuru Nairobi','Misc Nairobi',
                                    'Kilifi','Kisauni Mombasa','Misc Mombasa',
                                    'Nyanza','Turkana','other','Misc Rural Counties'])
mod_2_pivot = mod_2_pivot[['3:1','3:3','3:4','3:2']]
```

In [26]:

```
# module composition
filepath = os.path.join(figsdir,"modules.pdf")
fig, (ax1, ax2) = plt.subplots(1, 2, gridspec_kw={'width_ratios': [1, 1]}, sharey=True)

sns.heatmap(mod_1_pivot/mod_1_pivot.sum(),
            linewidths=0.5,
            cmap="Blues",
            vmin=0, 
            vmax=1,
            cbar=False,
            ax=ax1)

sns.heatmap(mod_2_pivot/mod_2_pivot.sum(),
            linewidths=0.5,
            cmap="Blues",
            vmin=0, 
            vmax=1,
            ax=ax2)

ax1.set_xlabel(None)
ax2.set_xlabel(None)
ax1.set_ylabel(None)
ax2.set_ylabel(None)
ax2.yaxis.set_visible(False)

ax1.tick_params(axis='both', which='major', labelsize=12)
ax2.tick_params(axis='both', which='major', labelsize=12)

plt.text(6, 7.4 ,"Fraction of module", fontsize=14, rotation=270)

plt.tight_layout()
plt.savefig(filepath)
plt.show()
```

### Business diversity¶

In [27]:

```
reg_users[reg_users['area_type']=='urban'][['id','business_type']].groupby('business_type').count().transpose()
```

Out[27]:

| business\_type | education | environment | faith | farming | food | fuel/energy | government | health | labour | other | savings | shop | system | transport | water |
| --- | --- | --- | --- | --- | --- | --- | --- | --- | --- | --- | --- | --- | --- | --- | --- |
| id | 291 | 181 | 14 | 100 | 4569 | 309 | 38 | 212 | 7626 | 661 | 172 | 2376 | 0 | 481 | 669 |

In [28]:

```
reg_users[reg_users['area_type']=='rural'][['id','business_type']].groupby('business_type').count().transpose()
```

Out[28]:

| business\_type | education | environment | faith | farming | food | fuel/energy | government | health | labour | other | savings | shop | system | transport | water |
| --- | --- | --- | --- | --- | --- | --- | --- | --- | --- | --- | --- | --- | --- | --- | --- |
| id | 116 | 49 | 8 | 7660 | 5056 | 2198 | 2 | 43 | 1871 | 662 | 85 | 2181 | 0 | 614 | 216 |

In [29]:

```
# size of the module an account is in
reg_users['mod_2_size'] = reg_users.groupby('mod_2')['id'].transform('count')
reg_users['mod_3_size'] = reg_users.groupby('mod_3')['id'].transform('count')
```

In [30]:

```
# business types in same/different accounts
reg_users['bus_2'] = reg_users['mod_2'].astype(str)+reg_users['business_type'].astype(str)
reg_users['bus_3'] = reg_users['mod_3'].astype(str)+reg_users['business_type'].astype(str)
reg_users['mod_2_sbus'] = reg_users.groupby('bus_2')['id'].transform('count')
reg_users['mod_3_sbus'] = reg_users.groupby('bus_3')['id'].transform('count')
reg_users['mod_2_dbus'] = reg_users['mod_2_size'] - reg_users['mod_2_sbus']
reg_users['mod_3_dbus'] = reg_users['mod_3_size'] - reg_users['mod_3_sbus']
reg_users['mod_2_fbus'] = reg_users['mod_2_dbus']/reg_users['mod_2_size']
reg_users['mod_3_fbus'] = reg_users['mod_3_dbus']/reg_users['mod_3_size']
reg_users = reg_users.drop(['bus_2','bus_3'],axis=1)
# and now geography, for comparison
reg_users['geo_2'] = reg_users['mod_2'].astype(str)+reg_users['area_name'].astype(str)
reg_users['geo_3'] = reg_users['mod_3'].astype(str)+reg_users['area_name'].astype(str)
reg_users['mod_2_sgeo'] = reg_users.groupby('geo_2')['id'].transform('count')
reg_users['mod_3_sgeo'] = reg_users.groupby('geo_3')['id'].transform('count')
reg_users['mod_2_dgeo'] = reg_users['mod_2_size'] - reg_users['mod_2_sgeo']
reg_users['mod_3_dgeo'] = reg_users['mod_3_size'] - reg_users['mod_3_sgeo']
reg_users['mod_2_fgeo'] = reg_users['mod_2_dgeo']/reg_users['mod_2_size']
reg_users['mod_3_fgeo'] = reg_users['mod_3_dgeo']/reg_users['mod_3_size']
reg_users = reg_users.drop(['geo_2','geo_3'],axis=1)
```

In [31]:

```
print("size")
print("averages:",reg_users['mod_2_size'].mean(), reg_users['mod_3_size'].mean())
print("mod 2", (reg_users['mod_2_size'].quantile(q=0.25),reg_users['mod_2_size'].quantile(q=0.75)))
print("mod 3", (reg_users['mod_3_size'].quantile(q=0.25),reg_users['mod_3_size'].quantile(q=0.75)))
```

```
size
averages: 1986.7010600880537 269.2301694665125
mod 2 (888.0, 3226.0)
mod 3 (26.0, 294.0)
```

In [32]:

```
print("business_type")
print("averages:",reg_users['mod_2_fbus'].mean(), reg_users['mod_3_fbus'].mean())
print("mod 2", (reg_users['mod_2_fbus'].quantile(q=0.25),reg_users['mod_2_fbus'].quantile(q=0.75)))
print("mod 3", (reg_users['mod_3_fbus'].quantile(q=0.25),reg_users['mod_3_fbus'].quantile(q=0.75)))
```

```
business_type
averages: 0.6613919439593097 0.5863725351503478
mod 2 (0.5373317013463892, 0.8474834773767158)
mod 3 (0.3701492537313433, 0.8076923076923077)
```

In [33]:

```
print("area_name")
print("averages:",reg_users['mod_2_fgeo'].mean(), reg_users['mod_3_fgeo'].mean())
print("mod 2", (reg_users['mod_2_fgeo'].quantile(q=0.25),reg_users['mod_2_fgeo'].quantile(q=0.75)))
print("mod 3", (reg_users['mod_3_fgeo'].quantile(q=0.25),reg_users['mod_3_fgeo'].quantile(q=0.75)))
```

```
area_name
averages: 0.14348031117404186 0.10143012814763203
mod 2 (0.006299580027998133, 0.13757115749525617)
mod 3 (0.0, 0.08484848484848485)
```

In [34]:

```
def plot_community_attributes(thenodes, directory, community_label='community', attribute_label='attr', max_communities=10, min_prevalence=10, prefix=''):
    plt.rcParams["axes.prop_cycle"] = plt.cycler("color", plt.cm.tab20(np.linspace(0,1,20)))
    thenodes = thenodes.reset_index()
    thenodes['attr_prevalence'] = thenodes.groupby(attribute_label)['node'].transform('count')
    thenodes = thenodes[~(thenodes['attr_prevalence'] <= min_prevalence)]  
    thenodes = thenodes[~(thenodes[community_label].isnull())]

    print(len(thenodes))
    communities = thenodes.groupby([community_label,attribute_label]).size().unstack()
    try:
        communities = communities.assign(total=communities.sum(axis=1))
    except:
        communities.columns = communities.columns.add_categories('total')
        communities = communities.assign(total=communities.sum(axis=1))
    # barh for horizontal, bar for vertical
    sortedcom = communities.sort_values(by='total',ascending=False)[0:max_communities].drop('total', axis=1)


    
    ax = sortedcom.plot.barh(stacked='True',figsize=(14,8))
    
    ax.set_xlabel("Nodes", fontsize=20)
    ax.set_ylabel("Sub-mobule", fontsize=20)
    
    ax.tick_params(axis='x', which='major', labelsize=18)
    ax.tick_params(axis='y', which='major', labelsize=4, labelcolor='w')
    
    # Hide the right and top spines
    ax.spines['right'].set_visible(False)
    ax.spines['top'].set_visible(False)
    
    lgd = plt.legend(loc='upper right', # bbox_to_anchor=(1.5, 1.05),
              ncol=4, frameon=False, shadow=True, fontsize=16) #fancybox=True

   
    labels = []
    for j in sortedcom.columns:
        for i in sortedcom.index:
            label = j#str(sortedcom.loc[i][j])
            labels.append(label)

    patches = ax.patches

   
    for label,rect in zip(labels, patches):
        # Find where everything is located
        height = rect.get_height()
        width = rect.get_width()
        x = rect.get_x()
        y = rect.get_y()

        # The height of the bar is the data value and can be used as the label
        label_text = label

        # ax.text(x, y, text)
        label_x = x + width / 2
        label_y = y + height / 2

        # only plot labels greater than given width
        if width > 400:
            ax.text(label_x, label_y, label_text, ha='center', va='center', fontsize=16)
    
    filename = prefix+community_label+"-"+attribute_label+".pdf"
    filepath = os.path.join(directory,filename)
    plt.tight_layout()
    plt.savefig(filepath)
    plt.show()
```

In [35]:

```
reg_users['area_name'] = reg_users['area_name'].cat.reorder_categories(['Kisauni Mombasa','Misc Mombasa','Turkana','other','Mukuru Nairobi','Misc Nairobi','Kilifi','Nyanza','Kinango Kwale','Misc Rural Counties'])
```

In [36]:

```
plot_community_attributes(reg_users, figsdir, community_label='mod_2', attribute_label='area_name', max_communities=15)
plot_community_attributes(reg_users, figsdir, community_label='mod_2', attribute_label='business_type', max_communities=15)
plot_community_attributes(reg_users, figsdir, community_label='mod_2', attribute_label='gender', max_communities=15)
plot_community_attributes(reg_users, figsdir, community_label='mod_2', attribute_label='prior', max_communities=15)
```

```
40657
```

```
40657
```

```
40657
```

```
40657
```

In [37]:

```
plot_community_attributes(reg_users, figsdir, community_label='mod_3', attribute_label='area_name', max_communities=25)
plot_community_attributes(reg_users, figsdir, community_label='mod_3', attribute_label='business_type', max_communities=25)
plot_community_attributes(reg_users, figsdir, community_label='mod_3', attribute_label='gender', max_communities=25)
plot_community_attributes(reg_users, figsdir, community_label='mod_3', attribute_label='prior', max_communities=25)
```

```
40657
```

```
40657
```

```
40657
```

```
40657
```

## Network stats¶

In [38]:

```
# undirected version that sums the edgeweights
flow_reg_undir = flow_reg_nx.to_undirected(flow_reg_nx)
# clear edge weights
for e in flow_reg_undir.edges():
    flow_reg_undir[e[0]][e[1]]['weight'] = 0
# sum edge weights
for e, d in flow_reg_nx.edges.items():
    flow_reg_undir[e[0]][e[1]]['weight'] += d['weight']
```

In [39]:

```
# network stats overall
flow_reg_dat = {}
flow_reg_dat["nodes"] = flow_reg_nx.number_of_nodes()    
flow_reg_dat["edges"] = flow_reg_nx.size()
flow_reg_dat["edges_undir"] = flow_reg_undir.size()
flow_reg_dat["volume"] = flow_reg_nx.size(weight="weight")
flow_reg_dat["density"] = nx.density(flow_reg_nx)
flow_reg_dat["density_undir"] = nx.density(flow_reg_undir)
```

In [40]:

```
# network stats by locality
flow_reg_dats = {}
for mod in mod_2s:
    flow_reg_dats[mod] = {}
    nodes = [node_id for node_id, attrs in flow_reg_nx.nodes.items() if attrs["mod_2"]==mod]
    tmp = flow_reg_nx.subgraph(nodes)
    tmp_undir = flow_reg_undir.subgraph(nodes)
    flow_reg_dats[mod]["nodes"] = tmp.number_of_nodes()    
    flow_reg_dats[mod]["edges"] = tmp.size()
    flow_reg_dats[mod]["edges_undir"] = tmp_undir.size()
    flow_reg_dats[mod]["volume"] = tmp.size(weight="weight")
    flow_reg_dats[mod]["density"] = nx.density(tmp)
    flow_reg_dats[mod]["density_undir"] = nx.density(tmp_undir)
```

### Assortativity¶

In [41]:

```
def attribute_assortativity(network,attribute,exclude=None):
    # avoid string-can-be-a-list issue
    if isinstance(exclude, str):
        exclude = [exclude]
    # filter by the known attributes
    if exclude is not None:
        nodes = [node_id for node_id, attrs in network.nodes.items() if attrs[attribute] not in exclude]
        tmp = network.subgraph(nodes)
    else:
        tmp = network
    # compute assortativity
    entries = set([attrs[attribute] for node_id, attrs in tmp.nodes.items()])
    if len(entries)==0:
        # not computable
        return np.nan
    elif len(entries)==1:
        # the networkx function gives nan for some reason
        return 1
    else:
        return nx.attribute_assortativity_coefficient(tmp,attribute)
```

In [42]:

```
import networkx.algorithms.community as nx_comm

# takes time to run modularity (for some reason)

def attribute_modularity(network,attribute,exclude=None):
    # avoid string-can-be-a-list issue
    if isinstance(exclude, str):
        exclude = [exclude]
    # filter by the known attributes
    if exclude is not None:
        nodes = [node_id for node_id, attrs in network.nodes.items() if attrs[attribute] not in exclude]
        tmp = network.subgraph(nodes)
    else:
        tmp = network
    # make communities of the attributes
    if exclude is None:
        values = set(dict(tmp.nodes(data=attribute)).values())
    else:
        values = set(dict(tmp.nodes(data=attribute)).values()) - set(exclude)
    communities = []
    for value in values:
        nodes = set([node_id for node_id, attrs in tmp.nodes.items() if attrs[attribute]==value])
        communities.append(nodes)
    # compute modularity
    try:
        mty = nx_comm.quality.modularity(tmp,communities,weight='weight')
    except:
        mty = float("nan")
    # return modularity
    return mty
```

#### Categoricals¶

In [43]:

```
for attribute, exclude in [('gender','Unknown'),
                           ('area_name','other'),
                           ('business_type','other'),
                           ('prior',None),
                           ('reg_month',None),
                           ('mod_1',None),
                           ('mod_2',None)]:
    # overall
    print(attribute)
    flow_reg_dat[attribute+"_aty"] = attribute_assortativity(flow_reg_nx,attribute,exclude=exclude)
    flow_reg_dat[attribute+"_aty_undir"] = attribute_assortativity(flow_reg_undir,attribute,exclude=exclude)
    flow_reg_dat[attribute+"_mty"] = attribute_modularity(flow_reg_nx,attribute,exclude=exclude)
    flow_reg_dat[attribute+"_mty_undir"] = attribute_modularity(flow_reg_undir,attribute,exclude=exclude)
    # by locality, for the empirical ones
    if attribute in ['mod_1','mod_2']:
        continue
    print("by module")
    for mod in mod_2s:
        nodes = [node_id for node_id, attrs in flow_reg_nx.nodes.items() if attrs["mod_2"]==mod]
        tmp = flow_reg_nx.subgraph(nodes)
        tmp_undir = flow_reg_undir.subgraph(nodes)
        # assortativity
        flow_reg_dats[mod][attribute+"_aty"] = attribute_assortativity(tmp,attribute,exclude=exclude) 
        flow_reg_dats[mod][attribute+"_aty_undir"] = attribute_assortativity(tmp_undir,attribute,exclude=exclude) 
        flow_reg_dats[mod][attribute+"_mty"] = attribute_modularity(tmp,attribute,exclude=exclude)
        flow_reg_dats[mod][attribute+"_mty_undir"] = attribute_modularity(tmp_undir,attribute,exclude=exclude)
```

```
gender
by module
area_name
by module
business_type
by module
prior
by module
reg_month
by module
mod_1
mod_2
```

##### Gender¶

In [44]:

```
usr_accts = [node_id for node_id, attrs in flow_reg_nx.nodes.items() if attrs['held_roles']!='GROUP_ACCOUNT']
```

In [45]:

```
# additional check on gender
flow_reg_dat["gender_aty_usr"] = attribute_assortativity(flow_reg_nx.subgraph(usr_accts),'gender',exclude=['Unknown'])
flow_reg_dat["gender_aty_usr_undir"] = attribute_assortativity(flow_reg_undir.subgraph(usr_accts),'gender',exclude=['Unknown'])
flow_reg_dat["gender_mty_usr"] = attribute_modularity(flow_reg_nx.subgraph(usr_accts),'gender',exclude=['Unknown'])
flow_reg_dat["gender_mty_usr_undir"] = attribute_modularity(flow_reg_undir.subgraph(usr_accts),'gender',exclude=['Unknown'])
for mod in mod_2s:
    nodes = [node_id for node_id, attrs in flow_reg_nx.nodes.items() if attrs["mod_2"]==mod]
    tmp = flow_reg_nx.subgraph(nodes)
    tmp_undir = flow_reg_undir.subgraph(nodes)
    flow_reg_dats[mod]["gender_aty_usr"] = attribute_assortativity(tmp.subgraph(usr_accts),'gender',exclude=['Unknown'])
    flow_reg_dats[mod]["gender_aty_usr_undir"] = attribute_assortativity(tmp_undir.subgraph(usr_accts),'gender',exclude=['Unknown'])
```

#### Numericals¶

In [46]:

```
import networkx.algorithms.link_analysis as nx_algo
```

In [47]:

```
flow_reg_n = flow_reg_nx.nodes()
# degree
nx.set_node_attributes(flow_reg_undir, {node:val for node, val in flow_reg_undir.degree() if node in flow_reg_n}, "deg")
nx.set_node_attributes(flow_reg_nx, {node:val for node, val in flow_reg_nx.in_degree() if node in flow_reg_n}, "deg_in")
nx.set_node_attributes(flow_reg_nx, {node:val for node, val in flow_reg_nx.out_degree() if node in flow_reg_n}, "deg_out")
# weighted degree
nx.set_node_attributes(flow_reg_undir, {node:val for node, val in flow_reg_undir.degree(weight='weight') if node in flow_reg_n}, "vol")
nx.set_node_attributes(flow_reg_nx, {node:val for node, val in flow_reg_nx.in_degree(weight='weight') if node in flow_reg_n}, "vol_in")
nx.set_node_attributes(flow_reg_nx, {node:val for node, val in flow_reg_nx.out_degree(weight='weight') if node in flow_reg_n}, "vol_out")
# admin-adjusted pagerank
admin_in = dict(flow_reg_nx.nodes(data="ovol_in"))
alpha=85
pagerank = nx_algo.pagerank(flow_reg_nx,weight="weight",alpha=alpha/100,personalization=admin_in)
nx.set_node_attributes(flow_reg_nx, pagerank, "pr_a"+str(alpha).zfill(3)+"_in")
```

In [48]:

```
for attribute in ['reg_age','sunique_in','sunique_out','deg_in','deg_out']:
    # overall
    flow_reg_dat[attribute+"_aty_n"] = nx.numeric_assortativity_coefficient(flow_reg_nx,attribute)
    # by locality
    for mod in mod_2s:
        nodes = [node_id for node_id, attrs in flow_reg_nx.nodes.items() if attrs["mod_2"]==mod]
        tmp = flow_reg_nx.subgraph(nodes)
        # assortativity
        flow_reg_dats[mod][attribute+"_aty_n"] = nx.numeric_assortativity_coefficient(tmp,attribute)
```

In [49]:

```
for attribute in ['reg_age','sunique_in','sunique_out','deg','vol']:
    # overall
    flow_reg_dat[attribute+"_aty_n_undir"] = nx.numeric_assortativity_coefficient(flow_reg_undir,attribute)
    # by locality
    for mod in mod_2s:
        nodes = [node_id for node_id, attrs in flow_reg_nx.nodes.items() if attrs["mod_2"]==mod]
        tmp = flow_reg_undir.subgraph(nodes)
        # assortativity
        flow_reg_dats[mod][attribute+"_aty_n_undir"] = nx.numeric_assortativity_coefficient(tmp,attribute)
```

#### Plot¶

In [50]:

```
flow_reg_dat
```

Out[50]:

```
{'nodes': 40657,
 'edges': 145661,
 'edges_undir': 107519,
 'volume': 293688301.0480015,
 'density': 8.812178988062824e-05,
 'density_undir': 0.00013009339117780693,
 'gender_aty': 0.19798380347535097,
 'gender_aty_undir': 0.1791445760557139,
 'gender_mty': 0.09008473693172256,
 'gender_mty_undir': 0.09006536087423926,
 'area_name_aty': 0.8576111893103525,
 'area_name_aty_undir': 0.8588209264180771,
 'area_name_mty': 0.43322584953280696,
 'area_name_mty_undir': 0.43322541995219965,
 'business_type_aty': 0.12454043940832436,
 'business_type_aty_undir': 0.12326950718391448,
 'business_type_mty': 0.012422990380832347,
 'business_type_mty_undir': 0.012123423239078689,
 'prior_aty': 0.2065977930598962,
 'prior_aty_undir': 0.18114229091597683,
 'prior_mty': 0.17392706195539528,
 'prior_mty_undir': 0.17378825192904196,
 'reg_month_aty': 0.2732872176724971,
 'reg_month_aty_undir': 0.2583482205069716,
 'reg_month_mty': 0.22344244595316123,
 'reg_month_mty_undir': 0.2234127608732833,
 'mod_1_aty': 0.9897625397741964,
 'mod_1_aty_undir': 0.9899501313513421,
 'mod_1_mty': 0.4763210490300102,
 'mod_1_mty_undir': 0.476321028115805,
 'mod_2_aty': 0.9231211036563035,
 'mod_2_aty_undir': 0.9204351472261187,
 'mod_2_mty': 0.8902463505224841,
 'mod_2_mty_undir': 0.8902417747763746,
 'gender_aty_usr': 0.19534430446284104,
 'gender_aty_usr_undir': 0.17538711101649096,
 'gender_mty_usr': 0.10227839674880242,
 'gender_mty_usr_undir': 0.10224555154411447,
 'reg_age_aty_n': 0.573546395053529,
 'sunique_in_aty_n': -0.0731044470050039,
 'sunique_out_aty_n': -0.05841696927607099,
 'deg_in_aty_n': -0.07297972022820975,
 'deg_out_aty_n': -0.058177762954114816,
 'reg_age_aty_n_undir': 0.5346015171117976,
 'sunique_in_aty_n_undir': -0.0787950484669324,
 'sunique_out_aty_n_undir': -0.05034257442988532,
 'deg_aty_n_undir': -0.07471264353150371,
 'vol_aty_n_undir': 0.009155977833053268}
```

In [51]:

```
# to pandas
flow_reg_dats_table = pd.DataFrame.from_dict(flow_reg_dats,orient='index')
flow_reg_dats_table = flow_reg_dats_table.rename_axis('mod_2').reset_index()
```

In [52]:

```
flow_reg_dats_table.describe()
```

Out[52]:

|  | nodes | edges | edges\_undir | volume | density | density\_undir | gender\_aty | gender\_aty\_undir | gender\_mty | gender\_mty\_undir | ... | reg\_age\_aty\_n | sunique\_in\_aty\_n | sunique\_out\_aty\_n | deg\_in\_aty\_n | deg\_out\_aty\_n | reg\_age\_aty\_n\_undir | sunique\_in\_aty\_n\_undir | sunique\_out\_aty\_n\_undir | deg\_aty\_n\_undir | vol\_aty\_n\_undir |
| --- | --- | --- | --- | --- | --- | --- | --- | --- | --- | --- | --- | --- | --- | --- | --- | --- | --- | --- | --- | --- | --- |
| count | 37.000000 | 37.000000 | 37.000000 | 3.700000e+01 | 37.000000 | 37.000000 | 37.000000 | 37.000000 | 36.000000 | 36.000000 | ... | 37.000000 | 37.000000 | 37.000000 | 37.000000 | 37.000000 | 37.000000 | 37.000000 | 37.000000 | 37.000000 | 37.000000 |
| mean | 1021.378378 | 3572.189189 | 2636.432432 | 7.666740e+06 | 0.008929 | 0.012062 | 0.165404 | 0.145695 | 0.038404 | 0.036748 | ... | 0.202862 | -0.173308 | -0.112638 | -0.173147 | -0.112127 | 0.154056 | -0.216254 | -0.124735 | -0.215015 | -0.066021 |
| std | 1081.676023 | 4796.264408 | 3788.618354 | 1.082685e+07 | 0.010160 | 0.012352 | 0.195915 | 0.188140 | 0.104866 | 0.103787 | ... | 0.259093 | 0.106824 | 0.124287 | 0.106773 | 0.124398 | 0.273029 | 0.121966 | 0.128488 | 0.119165 | 0.143060 |
| min | 136.000000 | 175.000000 | 170.000000 | 9.203000e+03 | 0.000712 | 0.000983 | -0.057895 | -0.080979 | -0.211856 | -0.211878 | ... | -0.213595 | -0.360006 | -0.288817 | -0.360006 | -0.285791 | -0.323111 | -0.452912 | -0.363779 | -0.447520 | -0.428028 |
| 25% | 222.000000 | 773.000000 | 544.000000 | 5.862862e+05 | 0.001666 | 0.002404 | 0.019881 | 0.017221 | -0.008608 | -0.008920 | ... | -0.004406 | -0.227507 | -0.192738 | -0.227507 | -0.192321 | -0.071710 | -0.266759 | -0.206655 | -0.264680 | -0.168378 |
| 50% | 537.000000 | 1695.000000 | 1151.000000 | 2.991597e+06 | 0.006021 | 0.009419 | 0.131149 | 0.120812 | 0.010494 | 0.010490 | ... | 0.197528 | -0.183284 | -0.142361 | -0.183257 | -0.141994 | 0.146582 | -0.217368 | -0.149628 | -0.221307 | -0.096209 |
| 75% | 1522.000000 | 4613.000000 | 3513.000000 | 1.105016e+07 | 0.011753 | 0.016759 | 0.242553 | 0.207741 | 0.071828 | 0.071121 | ... | 0.328068 | -0.125053 | -0.062569 | -0.124511 | -0.062510 | 0.321685 | -0.164800 | -0.078528 | -0.151844 | 0.011632 |
| max | 4286.000000 | 24816.000000 | 20458.000000 | 4.364446e+07 | 0.042529 | 0.051611 | 1.000000 | 1.000000 | 0.275579 | 0.273721 | ... | 0.892902 | 0.266792 | 0.278761 | 0.266792 | 0.281287 | 0.845417 | 0.286053 | 0.302912 | 0.246790 | 0.268903 |

8 rows × 38 columns

In [53]:

```
# save
os.makedirs(os.path.join(projdir,"analysis","communities"), exist_ok=True)
flow_reg_dats_fn = os.path.join(projdir,"analysis","communities","sarafu_reg_dats.csv")
flow_reg_dats_table.describe().to_csv(flow_reg_dats_fn)
```

In [ ]:

```

```
