# Supplementary material for: Circulation of a digital community currency
Source: Sci Rep. 2023 Apr 11;13:5864. doi: 10.1038/s41598-023-33184-1 (PMC10088680; doi:10.1038/s41598-023-33184-1)
Supplement: Supplementary file 3 — Supplementary Information 3. [file 41598_2023_33184_MOESM3_ESM.html]

SI\_3


In [1]:

```
import numpy as np
import pandas as pd
import json
import re
import os
import math
import random 
from collections import Counter
from datetime import datetime, timedelta
import networkx as nx
import matplotlib.pyplot as plt
import matplotlib.dates as mdates
import matplotlib as mpl
import seaborn as sns
%matplotlib inline
```

In [2]:

```
['#%02x%02x%02x' % (int(x[0]*255),int(x[1]*255),int(x[2]*255)) for x in list(sns.color_palette())]
```

Out[2]:

```
['#1f77b4',
 '#ff7f0e',
 '#2ca02c',
 '#d62728',
 '#9467bd',
 '#8c564b',
 '#e377c2',
 '#7f7f7f',
 '#bcbd22',
 '#17becf']
```

## Directory paths & data¶

In [3]:

```
# Define directories
homedir = os.path.expanduser("~")
datadir = os.path.join(homedir,'Documents','Research','Sarafu','Sarafu2021_UKDS')
projdir = os.path.join(homedir,'Documents','Research','Sarafu','Exploration')
figsdir = os.path.join(projdir,'figures')
```

#### Sarafu flow network¶

In [4]:

```
# Flow network w/ attributes
flow_reg_gexf = os.path.join(datadir,"networks","sarafu_reg_users.gexf")
flow_reg_nx = nx.read_gexf(flow_reg_gexf,relabel=True)
flow_reg_n = set(flow_reg_nx.nodes())
```

In [5]:

```
# Confirm the number of nodes & total volume
print("nodes",flow_reg_nx.number_of_nodes())
print("edges",flow_reg_nx.size())
print("volume",flow_reg_nx.size(weight="weight"))
```

```
nodes 40657
edges 145661
volume 293688301.0480015
```

### Centralities¶

In [6]:

```
import networkx.algorithms.link_analysis as nx_algo
```

#### Degree & weighted degree¶

In [7]:

```
# degree
nx.set_node_attributes(flow_reg_nx, {node:val for node, val in flow_reg_nx.in_degree() if node in flow_reg_n}, "deg_in")
nx.set_node_attributes(flow_reg_nx, {node:val for node, val in flow_reg_nx.out_degree() if node in flow_reg_n}, "deg_out")
```

In [8]:

```
# weighted degree
nx.set_node_attributes(flow_reg_nx, {node:val for node, val in flow_reg_nx.in_degree(weight='weight') if node in flow_reg_n}, "vol_in")
nx.set_node_attributes(flow_reg_nx, {node:val for node, val in flow_reg_nx.out_degree(weight='weight') if node in flow_reg_n}, "vol_out")
```

#### PageRank¶

In [9]:

```
# unweighted
for alpha in range(70,101):
    pagerank = nx_algo.pagerank(flow_reg_nx,weight=None,alpha=alpha/100)
    nx.set_node_attributes(flow_reg_nx, pagerank, "pr_a"+str(alpha).zfill(3))
```

In [10]:

```
# weighted
for alpha in range(70,101):
    pagerank = nx_algo.pagerank(flow_reg_nx,weight="weight",alpha=alpha/100)
    nx.set_node_attributes(flow_reg_nx, pagerank, "pr_a"+str(alpha).zfill(3)+"_w")
```

In [11]:

```
# admin-adjusted
admin_in = dict(flow_reg_nx.nodes(data="ovol_in"))
for alpha in range(70,101):
    try:
        pagerank = nx_algo.pagerank(flow_reg_nx,weight="weight",alpha=alpha/100,personalization=admin_in)
        nx.set_node_attributes(flow_reg_nx, pagerank, "pr_a"+str(alpha).zfill(3)+"_in")
    except:
        print(alpha)
```

##### Nodes¶

In [12]:

```
flow_reg_nodes = pd.DataFrame.from_dict(dict(flow_reg_nx.nodes(data=True)),orient='index')
# normalize values
for term in ["deg_in","deg_out","vol_in","vol_out"]:
    total = flow_reg_nodes[term].sum()
    flow_reg_nodes[term+"_norm"] = flow_reg_nodes[term].divide(total)
    nx.set_node_attributes(flow_reg_nx, flow_reg_nodes[term+"_norm"], term+"_norm")
```

In [13]:

```
flow_reg_nodes = flow_reg_nodes.reset_index()
```

### Correlations & plots¶

In [14]:

```
import seaborn as sns
from matplotlib.lines import Line2D
from scipy import stats
```

In [15]:

```
def get_sequence(network, node_attribute):
    # retrieve the node attribute
    return list(nx.get_node_attributes(network,node_attribute).values())
```

In [16]:

```
def get_distribution(data_sequence, number_of_bins = 30):
    # Modified from: https://github.com/ivanvoitalov/tail-estimation/
    # define the support of the distribution
    zeros = 0 in data_sequence
    lower_bound = min(data_sequence) if not zeros else min([k for k in data_sequence if k>0])
    upper_bound = max(data_sequence)
    # define bin edges
    log = np.log10
    upper_bound = log(upper_bound)
    lower_bound = log(lower_bound)
    bins = np.logspace(lower_bound, upper_bound, number_of_bins+1)
    if zeros:
        # construct a temporary bin for the zeros
        bins = np.insert(np.logspace(lower_bound, upper_bound, number_of_bins+1), 0, 0, axis=0)
    # compute the histogram using numpy
    y, _ = np.histogram(data_sequence, bins = bins, density = True)
    if zeros:
        # remove the temporary bin for the zeros, and the corresponding density
        bins = np.delete(bins, 0)
        y = np.delete(y, 0)
    # for each bin, compute its average
    x, _, _ = stats.binned_statistic(data_sequence, data_sequence, statistic='mean', bins = bins)
    # if bin is empty, drop it from the resulting list
    #drop_indices = [i for i,k in enumerate(y) if k == 0.0]
    #x = [k for i,k in enumerate(x) if i not in drop_indices]
    #y = [k for i,k in enumerate(y) if i not in drop_indices]
    return x, y
```

##### Binned degree distributions¶

In [17]:

```
filepath = os.path.join(figsdir,"pdf_degree.pdf")

# get PDF
in_x_pdf, in_y_pdf = get_distribution(get_sequence(flow_reg_nx,"deg_in"), number_of_bins = 18)
out_x_pdf, out_y_pdf = get_distribution(get_sequence(flow_reg_nx,"deg_out"), number_of_bins = 18)

# plot PDF
plot = plt.loglog(in_x_pdf, in_y_pdf, color = "#1f77b4", marker = "s",
                 lw = 1.5, markeredgecolor = "#1f77b4")
plot = plt.loglog(out_x_pdf, out_y_pdf, color = "#ff7f0e", marker = "s",
                 lw = 1.5, markeredgecolor = "#ff7f0e")

# labels
plt.xlabel(r"Unique transaction partners", fontsize = 16)
plt.ylabel(r"Probability density", fontsize = 16);
# custom legend
custom_lines = [Line2D([0], [0], color="#1f77b4", lw=2),
                Line2D([0], [0], color="#ff7f0e", lw=2)]
plt.legend(custom_lines, ['Degree (in)', 'Degree (out)'], frameon=False, fontsize=14)
# save
plt.savefig(filepath)
plt.tight_layout()
plt.show()
```

In [18]:

```
filepath = os.path.join(figsdir,"pdf_degree_norm.pdf")

# get PDF
in_x_pdf, in_y_pdf = get_distribution(get_sequence(flow_reg_nx,"deg_in_norm"), number_of_bins = 18)
out_x_pdf, out_y_pdf = get_distribution(get_sequence(flow_reg_nx,"deg_out_norm"), number_of_bins = 18)
pr_x_pdf, pr_y_pdf = get_distribution(get_sequence(flow_reg_nx,"pr_a085"), number_of_bins = 18)

# plot PDF
plot = plt.loglog(in_x_pdf, in_y_pdf, color = "#1f77b4", marker = "s",
                 lw = 1.5, markeredgecolor = "#1f77b4")
plot = plt.loglog(out_x_pdf, out_y_pdf, color = "#ff7f0e", marker = "s",
                 lw = 1.5, markeredgecolor = "#ff7f0e")
plot = plt.loglog(pr_x_pdf, pr_y_pdf, color = "#8c5a50", marker = "s",
                 lw = 1.5, markeredgecolor = "#8c5a50")

# labels
plt.xlabel(r"Centrality score", fontsize = 16)
plt.ylabel(r"Probability density", fontsize = 16);
# custom legend
custom_lines = [Line2D([0], [0], color="#1f77b4", lw=2),
                Line2D([0], [0], color="#ff7f0e", lw=2),
                Line2D([0], [0], color="#8c5a50", lw=2)]
plt.legend(custom_lines, ['Degree (in)', 'Degree (out)', 'Pagerank (a=0.85)'], frameon=False, fontsize=14)
# save
plt.savefig(filepath)
plt.tight_layout()
plt.show()
```

In [19]:

```
filepath = os.path.join(figsdir,"pdf_strength.pdf")

# get PDF
in_x_pdf, in_y_pdf = get_distribution(get_sequence(flow_reg_nx,"vol_in"), number_of_bins = 24)
out_x_pdf, out_y_pdf = get_distribution(get_sequence(flow_reg_nx,"vol_out"), number_of_bins = 24)

# plot PDF
plot = plt.loglog(in_x_pdf, in_y_pdf, color = "#1f77b4", marker = "s",
                 lw = 1.5, markeredgecolor = "#1f77b4")
plot = plt.loglog(out_x_pdf, out_y_pdf, color = "#ff7f0e", marker = "s",
                 lw = 1.5, markeredgecolor = "#ff7f0e")

# labels
plt.xlabel(r"Volume (Sarafu)", fontsize = 16)
plt.ylabel(r"Probability density", fontsize = 16);
# custom legend
custom_lines = [Line2D([0], [0], color="#1f77b4", lw=2),
                Line2D([0], [0], color="#ff7f0e", lw=2)]
plt.legend(custom_lines, ['W. Degree (in)', 'W. Degree (out)'], frameon=False, fontsize=14)
# save
plt.savefig(filepath)
plt.tight_layout()
plt.show()
```

In [20]:

```
filepath = os.path.join(figsdir,"pdf_strength_norm.pdf")

# get PDF
in_x_pdf, in_y_pdf = get_distribution(get_sequence(flow_reg_nx,"vol_in_norm"), number_of_bins = 18)
out_x_pdf, out_y_pdf = get_distribution(get_sequence(flow_reg_nx,"vol_out_norm"), number_of_bins = 18)
pr_x_pdf, pr_y_pdf = get_distribution(get_sequence(flow_reg_nx,"pr_a085_w"), number_of_bins = 18)
pr2_x_pdf, pr2_y_pdf = get_distribution(get_sequence(flow_reg_nx,"pr_a085_in"), number_of_bins = 18)

# plot PDF
plot = plt.loglog(in_x_pdf, in_y_pdf, color = "#1f77b4", marker = "s",
                 lw = 1.5, markeredgecolor = "#1f77b4")
plot = plt.loglog(out_x_pdf, out_y_pdf, color = "#ff7f0e", marker = "s",
                 lw = 1.5, markeredgecolor = "#ff7f0e")
plot = plt.loglog(pr_x_pdf, pr_y_pdf, color = "#8c5a50", marker = "s",
                 lw = 1.5, markeredgecolor = "#8c5a50")
plot = plt.loglog(pr2_x_pdf, pr2_y_pdf, color = "#555555", marker = "s",
                 lw = 1.5, markeredgecolor = "#555555")
# labels
plt.xlabel(r"Centrality score", fontsize = 16)
plt.ylabel(r"Probability density", fontsize = 16);
# custom legend
custom_lines = [Line2D([0], [0], color="#1f77b4", lw=2),
                Line2D([0], [0], color="#ff7f0e", lw=2),
                Line2D([0], [0], color="#8c5a50", lw=2),
                Line2D([0], [0], color="#555555", lw=2)]
plt.legend(custom_lines, ['W. Degree (in)', 'W. Degree (out)', 'W. Pagerank (a=0.85)', 'W. I. Pagerank (a=0.85)'], frameon=False, fontsize=14)
# save
plt.savefig(filepath)
plt.tight_layout()
plt.show()
```

#### Fitting the tail of the distribution¶

We estimate the tail exponent of these distributions with the method developed by Ivan Voitalov et al. (2019) using the software available in the accompanying GitHub repository. Briefly: Degree distributions that can be described by
$P(k)=\mathscr{l}(k) k^\gamma$, where $\mathscr{l}(k)$ is a slowly varying function, are so-called "regularly varying" distributions. We consider a distribution to have a power-law tail if it belongs to this class of distributions, that is, where $\frac{1}{\gamma-1}=\frac{1}{\alpha}=\xi>0$. The tail index $\xi$, the PDF tail exponent $\gamma$, and the CCDF tail exponent $\alpha$ can be estimated using proven, established estimators developed for extreme value theory. Please consult the cited paper and software for complete information on tail index estimation.

In [21]:

```
os.makedirs(os.path.join(projdir,"analysis","tail"), exist_ok=True)
```

In [22]:

```
def export_distribution(network, node_attribute, upweight=False, prefix=''):
    sequence_fn = os.path.join(datadir,"networks",prefix+node_attribute+'.seq')
    sequence = get_sequence(network,node_attribute)
    if upweight:
        factor = network.number_of_nodes()
        sequence = [factor*val for val in sequence]
    sequence_df = pd.DataFrame.from_dict(Counter(sequence), orient='index').reset_index()
    sequence_df.to_csv(sequence_fn, sep=" ", header=False, index=False)
```

In [23]:

```
# export distributions
for node_attribute in ["deg_in","deg_out","vol_in","vol_out"]:
    export_distribution(flow_reg_nx,node_attribute,prefix='sarafu_reg_')
```

In [24]:

```
# run tail-estimation using the following script, or via the command line:
```

```
#!/bin/bash

DATA='/Users/mattssonc/Documents/Research/Sarafu/Sarafu2021_UKDS/networks'
CODE='/Users/mattssonc/Documents/Research/GitHub/tail-estimation/Python3'
WORK='/Users/mattssonc/Documents/Research/Sarafu/Exploration/analysis/tail'

python $CODE/tail-estimation.py $DATA/sarafu_reg_deg_in.seq $WORK/deg_in_fit.pdf --diagplots 1 > $WORK/deg_in_fit.txt
python $CODE/tail-estimation.py $DATA/sarafu_reg_deg_out.seq $WORK/deg_out_fit.pdf --diagplots 1 > $WORK/deg_out_fit.txt
python $CODE/tail-estimation.py $DATA/sarafu_reg_vol_in.seq $WORK/vol_in_fit.pdf  --diagplots 1 > $WORK/vol_in_fit.txt
python $CODE/tail-estimation.py $DATA/sarafu_reg_vol_in.seq $WORK/vol_in_fit.pdf  --diagplots 1 > $WORK/vol_out_fit.txt
```

In [25]:

```
# Load the tail-estimation results
gamma_fits = []
for node_attribute in ["deg_in","deg_out","vol_in","vol_out"]:
    gamma_fit_fn = os.path.join(projdir,"analysis","tail",node_attribute+'_fit.txt')
    gamma_fit = {}
    gamma_fit['attr'] = node_attribute
    with open(gamma_fit_fn,'r') as gamma_fit_txt:
        for line in gamma_fit_txt:
            if ' estimated gamma: ' in line:
                estimate = line.strip().split(' estimated gamma: ')
                gamma_fit[estimate[0]] = estimate[1]
    gamma_fits.append(gamma_fit)
# into a dataframe
gamma_fits = pd.DataFrame.from_dict(gamma_fits)
gamma_fits = gamma_fits.set_index('attr')
```

##### Degree¶

In [26]:

```
gamma_fits.loc[['deg_in','deg_out'],:]
```

Out[26]:

|  | Adjusted Hill | Moments | Kernel-type |
| --- | --- | --- | --- |
| attr |  |  |  |
| deg\_in | 2.908313858829886 | 2.884987476455898 | 3.7329510844543194 |
| deg\_out | 2.2191950309453596 | 2.9357202207088573 | 2.2051767931594997 |

By consulting the diagnostic plots, we conclude that the best estimate of the PDF tail exponent is ~2.9 for the in- and out- degree. The files `deg_in_fit.pdf` and `deg_out_fit.pdf` include plots of the CCDF tail-estimates against the empirical CCDF; those with a CCDF tail exponent of ~1.9 best fit the tail in both cases. Moreover, the files `deg_in_fit_diag.pdf` and `deg_out_fit_diag.pdf` illustrate the minimization used to select the best fit. The Moments estimator appears well-behaved in both cases and consistently selects a cutoff-point where 3% of the nodes are included in the tail of the in-degree distribution and 1% for the out-degree distribution; this corresponds to a PDF tail exponent ~2.9. Notably, the other estimators also produce clear minima at or near this cutoff-point. Lowering the tail cutoff to include the bulk of the out-degree distribution produces a lower estimate for the PDF tail exponent, at around 2.2.

##### Weighted degree¶

In [27]:

```
gamma_fits.loc[['vol_in','vol_out'],:]
```

Out[27]:

|  | Adjusted Hill | Moments | Kernel-type |
| --- | --- | --- | --- |
| attr |  |  |  |
| vol\_in | 3.2612679819201866 | 4.713671043400444 | 2.54980866958007 |
| vol\_out | 3.3278662702825734 | 4.557046882046343 | 2.5290322564723553 |

In contrast, the tail exponents for the weighted degree distributions are inconsistently estimated. The files `vol_in_fit.pdf` and `vol_out_fit.pdf` include plots of the CCDF tail-estimates against the empirical CCDF; the tail exponent depends strongly on what fraction of the nodes are included in the tail. This is reflected in files `vol_in_fit_diag.pdf` and `vol_out_fit_diag.pdf` as the lack of clear minima able to anchor the estimators.

#### Correlations¶

In [28]:

```
# adjust the final balance
flow_reg_nodes.loc[flow_reg_nodes['final_bal']=='','final_bal'] = 'nan'
flow_reg_nodes['final_bal'] = flow_reg_nodes['final_bal'].astype(float)
```

In [29]:

```
# pre-computed vs. computed -- not much of an impact from removing system accounts
print('  in degree',np.corrcoef(flow_reg_nodes["sunique_in"],flow_reg_nodes['deg_in'])[1,0])
print(' out degree',np.corrcoef(flow_reg_nodes["sunique_out"],flow_reg_nodes['deg_out'])[1,0])
print('  in volume',np.corrcoef(flow_reg_nodes["svol_in"],flow_reg_nodes['vol_in'])[1,0])
print(' out volume',np.corrcoef(flow_reg_nodes["svol_out"],flow_reg_nodes['vol_out'])[1,0])
```

```
  in degree 0.9999816667006289
 out degree 0.9999389238469881
  in volume 0.9999680961418054
 out volume 0.9992516608827742
```

In [30]:

```
# degree vs. pageranks
print('base       ',np.corrcoef(flow_reg_nodes["sunique_in"],flow_reg_nodes['sunique_out'])[1,0])
print('in  vs 0.7 ',np.corrcoef(flow_reg_nodes["sunique_in"],flow_reg_nodes['pr_a070'])[1,0])
print('out vs 0.7 ',np.corrcoef(flow_reg_nodes["sunique_out"],flow_reg_nodes['pr_a070'])[1,0])
print('in  vs 0.85',np.corrcoef(flow_reg_nodes["sunique_in"],flow_reg_nodes['pr_a085'])[1,0])
print('out vs 0.85',np.corrcoef(flow_reg_nodes["sunique_out"],flow_reg_nodes['pr_a085'])[1,0])
print('in  vs 1.0 ',np.corrcoef(flow_reg_nodes["sunique_in"],flow_reg_nodes['pr_a100'])[1,0])
print('out vs 1.0 ',np.corrcoef(flow_reg_nodes["sunique_out"],flow_reg_nodes['pr_a100'])[1,0])
```

```
base        0.8573778371121445
in  vs 0.7  0.882770035287776
out vs 0.7  0.7509488739005011
in  vs 0.85 0.8681950315763716
out vs 0.85 0.7364575807441524
in  vs 1.0  0.8006606025511989
out vs 1.0  0.6704933960890603
```

In [31]:

```
# weighted degree vs. pageranks
print('base       ',np.corrcoef(flow_reg_nodes["svol_in"],flow_reg_nodes['svol_out'])[1,0])
print('in  vs 0.7 ',np.corrcoef(flow_reg_nodes["svol_in"],flow_reg_nodes['pr_a070_w'])[1,0])
print('out vs 0.7 ',np.corrcoef(flow_reg_nodes["svol_out"],flow_reg_nodes['pr_a070_w'])[1,0])
print('in  vs 0.85',np.corrcoef(flow_reg_nodes["svol_in"],flow_reg_nodes['pr_a085_w'])[1,0])
print('out vs 0.85',np.corrcoef(flow_reg_nodes["svol_out"],flow_reg_nodes['pr_a085_w'])[1,0])
print('in  vs 1.0 ',np.corrcoef(flow_reg_nodes["svol_in"],flow_reg_nodes['pr_a100_w'])[1,0])
print('out vs 1.0 ',np.corrcoef(flow_reg_nodes["svol_out"],flow_reg_nodes['pr_a100_w'])[1,0])
```

```
base        0.9968523796161344
in  vs 0.7  0.6323996435673093
out vs 0.7  0.6092812272627962
in  vs 0.85 0.6532707638357046
out vs 0.85 0.6279442297559014
in  vs 1.0  0.6623749054187391
out vs 1.0  0.6379684821129442
```

In [32]:

```
# weighted degree w/ inflows vs. pageranks
print('base       ',np.corrcoef(flow_reg_nodes["svol_in"],flow_reg_nodes['svol_out'])[1,0])
print('in  vs 0.7 ',np.corrcoef(flow_reg_nodes["svol_in"],flow_reg_nodes['pr_a070_in'])[1,0])
print('out vs 0.7 ',np.corrcoef(flow_reg_nodes["svol_out"],flow_reg_nodes['pr_a070_in'])[1,0])
print('in  vs 0.85',np.corrcoef(flow_reg_nodes["svol_in"],flow_reg_nodes['pr_a085_in'])[1,0])
print('out vs 0.85',np.corrcoef(flow_reg_nodes["svol_out"],flow_reg_nodes['pr_a085_in'])[1,0])
print('in  vs 1.0 ',np.corrcoef(flow_reg_nodes["svol_in"],flow_reg_nodes['pr_a100_in'])[1,0])
print('out vs 1.0 ',np.corrcoef(flow_reg_nodes["svol_out"],flow_reg_nodes['pr_a100_in'])[1,0])
```

```
base        0.9968523796161344
in  vs 0.7  0.662523612843386
out vs 0.7  0.6404956819604892
in  vs 0.85 0.6816620538893625
out vs 0.85 0.6571834973858431
in  vs 1.0  0.6694167993299434
out vs 1.0  0.6449228468615285
```

In [33]:

```
flow_reg_nodes.columns
```

Out[33]:

```
Index(['index', 'final_bal', 'gender', 'area_name', 'area_type', 'held_roles',
       'business_type', 'old_POA_blockchain_address', 'ovol_in', 'ovol_out',
       ...
       'pr_a095_in', 'pr_a096_in', 'pr_a097_in', 'pr_a098_in', 'pr_a099_in',
       'pr_a100_in', 'deg_in_norm', 'deg_out_norm', 'vol_in_norm',
       'vol_out_norm'],
      dtype='object', length=136)
```

In [34]:

```
# correlations as a plot
flow_reg_corr = flow_reg_nodes[['deg_in','deg_out','pr_a085','vol_in','vol_out','pr_a085_w','pr_a085_in']].corr(method='pearson')
# upper triangular mask 
mask = np.triu(np.ones_like(flow_reg_corr, dtype=bool))
```

In [35]:

```
# module composition
filepath = os.path.join(figsdir,"centralities.pdf")
fig, ax = plt.subplots()

sns.heatmap(flow_reg_corr,
            mask = mask,
            linewidths=0.5,
            annot=True,
            cmap="Blues",
            vmin=0, 
            vmax=1,
            ax=ax)

ax.xaxis.set_visible(False)
ax.tick_params(axis='both', which='major', labelsize=12)

for tick in ax.yaxis.get_major_ticks():
    tick.tick1line.set_visible(False)
    tick.tick2line.set_visible(False)

ax.set_yticklabels(['','Degree (out)','PageRank','W. Degree (in)','W. Degree (out)','W. PageRank','W. I. PageRank'])
    
plt.text(0.4, 0.8,"Degree (in)", fontsize=12, rotation=45)
plt.text(1.4, 1.8,"Degree (out)", fontsize=12, rotation=45)
plt.text(2.4, 2.8,"PageRank", fontsize=12, rotation=45)
plt.text(3.4, 3.8,"W. Degree (in)", fontsize=12, rotation=45)
plt.text(4.4, 4.8,"W. Degree (out)", fontsize=12, rotation=45)
plt.text(5.4, 5.8,"W. PageRank", fontsize=12, rotation=45)

plt.text(8.6, 5.3,"Pearson correlation", fontsize=14, rotation=270)

plt.tight_layout()
plt.savefig(filepath)
plt.show()
```

In [36]:

```
# now vs final balance
tmp = flow_reg_nodes[~flow_reg_nodes['final_bal'].isna()].copy()
```

In [37]:

```
# pre-computed
tmp["computed"] = tmp["svol_in"]+tmp["ovol_in"]-tmp["svol_out"]-tmp["ovol_out"]
```

In [38]:

```
# degree & weighted degree vs. final balance
print(np.corrcoef(tmp["sunique_in"],tmp['final_bal'])[1,0])
print(np.corrcoef(tmp["sunique_out"],tmp['final_bal'])[1,0])
print(np.corrcoef(tmp["svol_in"],tmp['final_bal'])[1,0])
print(np.corrcoef(tmp["svol_out"],tmp['final_bal'])[1,0])
print(np.corrcoef(tmp["ovol_in"],tmp['final_bal'])[1,0])
print(np.corrcoef(tmp["computed"],tmp['final_bal'])[1,0])
```

```
0.2820961966408953
0.20507998842829175
0.521181762355547
0.4666535861573097
0.29239291589772193
0.9999994485221652
```

In [39]:

```
# pagerank vs final balance
pr_bal = pd.DataFrame({"term":np.array(range(70,101))})
pr_bal['alpha'] = pr_bal['term'].apply(lambda a: a/100)
pr_bal['restart'] = pr_bal['alpha'].apply(lambda a: 1-a)
pr_bal['pagerank_w'] = pr_bal['term'].apply(lambda a: np.corrcoef(tmp["pr_a%03d_w" % a],tmp['final_bal'])[1,0])
pr_bal['pagerank_in'] = pr_bal['term'].apply(lambda a: np.corrcoef(tmp["pr_a%03d_in" % a],tmp['final_bal'])[1,0])
```

In [40]:

```
# scatter
filepath = os.path.join(figsdir,"pageranks.pdf")
fig, ax = plt.subplots()

sns.scatterplot(data=pr_bal,
            x="alpha",
            y="pagerank_in",
            label="Weighted Inflow-adjusted PageRank",
            ax=ax)

sns.scatterplot(data=pr_bal,
            x="alpha",
            y="pagerank_w",
            label="Weighted PageRank",
            ax=ax)

ax.set_xlabel("Parameter alpha", fontsize=16)
ax.set_ylabel("Correlation", fontsize=16)
#ax.set_ylim(0.515,0.575)

ax.tick_params(axis='both', which='major', labelsize=12)

# Hide the right and top spines
ax.spines['right'].set_visible(False)
ax.spines['top'].set_visible(False)

sns.move_legend(ax, "lower left", frameon=False, fontsize=14)

plt.tight_layout()
plt.savefig(filepath)
plt.show()
```

### Regressions¶

In [41]:

```
from sklearn.linear_model import ElasticNet
from statsmodels.formula.api import ols
from sklearn.metrics import mean_squared_error
```

In [42]:

```
import statsmodels as sm
sm.__version__
```

Out[42]:

```
'0.13.2'
```

#### Ordering the categoricals¶

In [43]:

```
flow_reg_nodes.columns
```

Out[43]:

```
Index(['index', 'final_bal', 'gender', 'area_name', 'area_type', 'held_roles',
       'business_type', 'old_POA_blockchain_address', 'ovol_in', 'ovol_out',
       ...
       'pr_a095_in', 'pr_a096_in', 'pr_a097_in', 'pr_a098_in', 'pr_a099_in',
       'pr_a100_in', 'deg_in_norm', 'deg_out_norm', 'vol_in_norm',
       'vol_out_norm'],
      dtype='object', length=136)
```

In [44]:

```
# re-order area reference category to the largest -- "Kinango Kwale"
flow_reg_nodes['area_name'] = flow_reg_nodes['area_name'].astype("category").cat.reorder_categories([
    'Kinango Kwale','Mukuru Nairobi', 'Misc Nairobi', 'Kisauni Mombasa','Misc Mombasa','Kilifi',
    'Nyanza','Turkana','Misc Rural Counties','other'], ordered=False)
flow_reg_nodes['area_name'].value_counts()
```

Out[44]:

```
Kinango Kwale          19755
Mukuru Nairobi          8532
Kisauni Mombasa         5842
Misc Nairobi            2760
other                   1564
Kilifi                   633
Nyanza                   587
Misc Mombasa             565
Misc Rural Counties      213
Turkana                  206
Name: area_name, dtype: int64
```

In [45]:

```
# re-order business reference category to the largest in both urban/rural -- "food"
flow_reg_nodes['business_type'] = flow_reg_nodes['business_type'].astype("category").cat.reorder_categories([
    'food', 'shop', 'labour', 'savings', 'fuel/energy', 'other', 'transport', 'health', 
    'water', 'farming', 'education','government', 'environment', 'faith'], ordered=False)
flow_reg_nodes['business_type'].value_counts()
```

Out[45]:

```
labour         11205
food            9783
farming         7811
shop            4613
fuel/energy     2527
other           1395
transport       1134
water            893
education        469
savings          264
health           261
environment      234
government        42
faith             26
Name: business_type, dtype: int64
```

In [46]:

```
# re-roder gender reference category to the neutral one -- "Unknown"
flow_reg_nodes['gender'] = flow_reg_nodes['gender'].astype("category").cat.reorder_categories([
    'Unknown', 'Male', 'Female', 'Other'], ordered=False)
flow_reg_nodes['gender'].value_counts()
```

Out[46]:

```
Unknown    16903
Male       14130
Female      9603
Other         21
Name: gender, dtype: int64
```

In [47]:

```
flow_reg_nodes['prior'].value_counts()
```

Out[47]:

```
False    37252
True      3405
Name: prior, dtype: int64
```

In [48]:

```
flow_reg_nodes["pr_a085_in_ln"] = np.log(flow_reg_nodes["pr_a085_in"])
flow_reg_nodes["pr_a085_ln"] = np.log(flow_reg_nodes["pr_a085"])
```

#### Select the penalty weight|¶

In [49]:

```
# implement cross-validation
def cv(dataset, formula, alpha, cv_col='fold', L1_wt=0.5):
    # output metric is MSE & pearson correlation (r2)
    row = {'alpha':alpha}
    row['outcome'] = formula.split('~')[0].strip()
    for fold in dataset[cv_col].unique():
        # fit model 
        model = ols(formula, data=dataset[dataset[cv_col]!=fold])
        fit = model.fit_regularized(alpha=alpha,L1_wt=L1_wt)
        # predict unseen fold
        pred_vals = fit.predict(exog=dataset[[term.strip(' )C(') for term in formula.split('~')[1].strip(' )C(').split('+')]][dataset[cv_col]==fold])
        # grab actual values
        real_vals = dataset[[formula.split('~')[0].strip()]][dataset[cv_col]==fold].squeeze()
        # store the mse
        row[str(fold)+"mse"] = mean_squared_error(y_true=real_vals,y_pred=pred_vals)
        # store the number of variables
        row[str(fold)+"vars"] = sum(fit.params!=0)
    # return
    return row
```

In [50]:

```
# implement the loop over alphas
def loop_alphas(dataset,formula,folds,alphas,L1_wt=0.5):
    # set the seed
    random.seed(505)
    # cross-validation folds
    dataset['fold'] = random.choices(list(range(folds)), k=len(dataset.index))
    # initialize output
    output = []
    # loop alphas
    for alpha in alphas:
        output.append(cv(dataset,formula,alpha))
    # join into a df
    return pd.DataFrame(output)
```

##### Run¶

In [51]:

```
models_cvs = {}
models = {}
```

In [52]:

```
results = {}
```

In [53]:

```
alphas = [10**(-num/10) for num in range(10,100,1)]
regressors = 'reg_age + C(prior) + C(area_name) + C(business_type) + C(gender)'
```

In [54]:

```
# models
for outcome in ['pr_a085','pr_a085_in']:
    models_cvs[outcome] = loop_alphas(flow_reg_nodes," ~ ".join([outcome,regressors]),5,alphas,L1_wt=0.5)
```

In [55]:

```
# models
for outcome in ['pr_a085','pr_a085_in']:
    models_cvs[outcome]['mse'] = models_cvs[outcome][['0mse','1mse','2mse','3mse','4mse']].mean(axis=1)
    models_cvs[outcome]['vars']  = models_cvs[outcome][['0vars','1vars','2vars','3vars','4vars']].mean(axis=1)
```

In [56]:

```
# scatter
filepath = os.path.join(figsdir,"alphas_pr_a085_in.pdf")
fig, ax = plt.subplots()

sns.scatterplot(data=models_cvs['pr_a085_in'],
            x="alpha",
            y="mse",
            color=sns.color_palette("Paired")[1],
            ax=ax)

ax2 = ax.twinx()

sns.scatterplot(data=models_cvs['pr_a085_in'],
            x="alpha",
            y="vars",
            color=sns.color_palette("Paired")[0],
            ax=ax2)

ax.set_xscale("log")
ax.invert_xaxis()
ax.set_xlabel("Penalty weight")
ax.set_ylabel("Mean Squared Error")
ax2.set_ylabel("Number of parameters")
#ax.set_ylim(0.515,0.575)

#sns.move_legend(ax, "center left", frameon=False)
#sns.move_legend(ax2, "center right", frameon=False)


plt.tight_layout()
plt.savefig(filepath)
plt.show()
```

In [57]:

```
models_cvs['pr_a085_in'][models_cvs['pr_a085_in']['vars']==7]['alpha'].values
```

Out[57]:

```
array([2.51188643e-06, 1.99526231e-06, 1.58489319e-06, 1.25892541e-06])
```

In [58]:

```
model = ols('pr_a085_in ~ reg_age + C(prior) + C(area_name) + C(business_type) + C(gender)', data=flow_reg_nodes)
models["pr_a085_in_reg"] = model.fit_regularized(alpha=1.25892541e-06,L1_wt=0.5)
```

In [59]:

```
model = ols('pr_a085_in ~ reg_age + C(prior) + C(area_name) + C(business_type) + C(gender)', data=flow_reg_nodes)
models["pr_a085_in"] = model.fit()
```

In [60]:

```
# OLS
params = pd.DataFrame(models["pr_a085_in"].params).reset_index()
params.columns = ["var","coef"]
# variable names 
params["variable"] = params["var"].apply(lambda x: x.strip("C()[]").split(")[T.")[0])
params["value"] = params["var"].apply(lambda x: x.strip("C()[]").split(")[T.")[-1])
params.loc[params["value"]=="True","value"] = "Prior account"
params.loc[params["value"]=="reg_age","value"] = "Account age"
params.loc[params["value"]=="other","value"] = "Unknown"
lowercase = params["value"].apply(lambda x: x.islower())
params.loc[lowercase,"value"] = params.loc[lowercase,"value"].apply(lambda x: x.capitalize())
params["outcome"] = "W. I. PageRank, OLS"
reg_age = params.iloc[-1]
params = params.shift(1)
params.iloc[0] = params.iloc[1]
params.iloc[1] = reg_age
# lower confidence interval (will become line)
tmp = pd.DataFrame([interval[0] for interval in models["pr_a085_in"].conf_int().values])
tmp.columns = ["coef"]
reg_age = tmp.iloc[-1]
tmp = tmp.shift(1)
tmp.iloc[0] = tmp.iloc[1]
tmp.iloc[1] = reg_age
tmp["variable"] = params["variable"]
tmp["value"] = params["value"]
tmp["outcome"] = "W. I. PageRank, OLS"
results["pr_a085_in"] = pd.concat([params,tmp])
# upper confidence interval (will become line)
tmp = pd.DataFrame([interval[1] for interval in models["pr_a085_in"].conf_int().values])
tmp.columns = ["coef"]
reg_age = tmp.iloc[-1]
tmp = tmp.shift(1)
tmp.iloc[0] = tmp.iloc[1]
tmp.iloc[1] = reg_age
tmp["variable"] = params["variable"]
tmp["value"] = params["value"]
tmp["outcome"] = "W. I. PageRank, OLS"
results["pr_a085_in"] = pd.concat([results["pr_a085_in"],tmp])

# regularized
tmp = pd.DataFrame(models["pr_a085_in_reg"].params.values)
tmp.columns = ["coef"]
reg_age = tmp.iloc[-1]
tmp = tmp.shift(1)
tmp.iloc[0] = tmp.iloc[1]
tmp.iloc[1] = reg_age
tmp["variable"] = params["variable"]
tmp["value"] = params["value"]
tmp["outcome"] = "W. I. PageRank, EN"
results["pr_a085_in"] = pd.concat([results["pr_a085_in"],tmp])
# remove zeros
results["pr_a085_in"].loc[results["pr_a085_in"]["coef"]==0,"coef"] = np.nan
```

#### Unweighted PageRank¶

In [61]:

```
# scatter
filepath = os.path.join(figsdir,"alphas_pr_a085.pdf")
fig, ax = plt.subplots()

sns.scatterplot(data=models_cvs['pr_a085'],
            x="alpha",
            y="mse",
            color=sns.color_palette("Paired")[1],
            ax=ax)

ax2 = ax.twinx()

sns.scatterplot(data=models_cvs['pr_a085'],
            x="alpha",
            y="vars",
            color=sns.color_palette("Paired")[0],
            ax=ax2)

ax.set_xscale("log")
ax.invert_xaxis()
ax.set_xlabel("Penalty weight")
ax.set_ylabel("Mean Squared Error")
ax2.set_ylabel("Number of parameters")
#ax.set_ylim(0.515,0.575)

#sns.move_legend(ax, "center left", frameon=False)
#sns.move_legend(ax2, "center right", frameon=False)


plt.tight_layout()
plt.savefig(filepath)
plt.show()
```

In [62]:

```
models_cvs['pr_a085'][models_cvs['pr_a085']['vars']==5]['alpha'].values
```

Out[62]:

```
array([2.51188643e-06, 1.99526231e-06])
```

In [63]:

```
model = ols('pr_a085 ~ reg_age + C(prior) + C(area_name) + C(business_type) + C(gender)', data=flow_reg_nodes)
models["pr_a085_reg"] = model.fit_regularized(alpha=1.99526231e-06,L1_wt=0.5)
```

In [64]:

```
model = ols('pr_a085 ~ reg_age + C(prior) + C(area_name) + C(business_type) + C(gender)', data=flow_reg_nodes)
models["pr_a085"] = model.fit()
```

In [65]:

```
# OLS
params = pd.DataFrame(models["pr_a085"].params).reset_index()
params.columns = ["var","coef"]
# variable names 
params["variable"] = params["var"].apply(lambda x: x.strip("C()[]").split(")[T.")[0])
params["value"] = params["var"].apply(lambda x: x.strip("C()[]").split(")[T.")[-1])
params.loc[params["value"]=="True","value"] = "Prior account"
params.loc[params["value"]=="reg_age","value"] = "Account age"
params.loc[params["value"]=="other","value"] = "Unknown"
lowercase = params["value"].apply(lambda x: x.islower())
params.loc[lowercase,"value"] = params.loc[lowercase,"value"].apply(lambda x: x.capitalize())
params["outcome"] = "PageRank, OLS"
reg_age = params.iloc[-1]
params = params.shift(1)
params.iloc[0] = params.iloc[1]
params.iloc[1] = reg_age
# lower confidence interval (will become line)
tmp = pd.DataFrame([interval[0] for interval in models["pr_a085"].conf_int().values])
tmp.columns = ["coef"]
reg_age = tmp.iloc[-1]
tmp = tmp.shift(1)
tmp.iloc[0] = tmp.iloc[1]
tmp.iloc[1] = reg_age
tmp["variable"] = params["variable"]
tmp["value"] = params["value"]
tmp["outcome"] = "PageRank, OLS"
results["pr_a085"] = pd.concat([params,tmp])
# upper confidence interval (will become line)
tmp = pd.DataFrame([interval[1] for interval in models["pr_a085"].conf_int().values])
tmp.columns = ["coef"]
reg_age = tmp.iloc[-1]
tmp = tmp.shift(1)
tmp.iloc[0] = tmp.iloc[1]
tmp.iloc[1] = reg_age
tmp["variable"] = params["variable"]
tmp["value"] = params["value"]
tmp["outcome"] = "PageRank, OLS"
results["pr_a085"] = pd.concat([results["pr_a085"],tmp])

# regularized
tmp = pd.DataFrame(models["pr_a085_reg"].params.values)
tmp.columns = ["coef"]
reg_age = tmp.iloc[-1]
tmp = tmp.shift(1)
tmp.iloc[0] = tmp.iloc[1]
tmp.iloc[1] = reg_age
tmp["variable"] = params["variable"]
tmp["value"] = params["value"]
tmp["outcome"] = "PageRank, EN"
results["pr_a085"] = pd.concat([results["pr_a085"],tmp])
# remove zeros
results["pr_a085"].loc[results["pr_a085"]["coef"]==0,"coef"] = np.nan
```

In [66]:

```
# Plot
to_plot = pd.concat([results["pr_a085_in"],results["pr_a085"]])
to_plot["outcome"] = to_plot["outcome"].astype("category").cat.reorder_categories([
    'PageRank, OLS', 'W. I. PageRank, OLS', 'PageRank, EN', 'W. I. PageRank, EN'], ordered=False)

filepath = os.path.join(figsdir,"coefs_pr_a085.pdf")
fig, ax = plt.subplots(figsize=(8,8))

ax.axvline(x=0,linestyle='--',linewidth=1,color="grey",zorder=0)

colors = [sns.color_palette("Paired")[i] for i in [2,0,3,1]]

sns.pointplot(x='coef', y='value', hue='outcome', palette=colors, estimator=np.median, ci=100,
    data=to_plot, dodge=True, join=False, markers=["o","o","d","d"], fontsize=12, ax=ax)

ax.set_xlabel("Coefficient", fontsize=12)
ax.set_ylabel(None)
ax.tick_params(axis='y', which='major', labelsize=12)
ax.set_xlim(-10**(-3),10**(-3))

# Bump legend right 
legend_order = [0,2,1,3]
handles, labels = ax.get_legend_handles_labels()
plt.legend([handles[idx] for idx in legend_order],[labels[idx] for idx in legend_order],
          loc=1, title=None, fontsize=12, frameon=False)

fig.text(0.14, 0.56, "---Geographic area---", fontsize=12, rotation=270)
fig.text(0.14, 0.222, "--------Livelihood category-------", fontsize=12, rotation=270)
fig.text(0.14, 0.135, "Gender", fontsize=12, rotation=270)

plt.savefig(filepath, bbox_inches='tight')
plt.show()
```

In [ ]:

```

```
