# Supplementary material for: Circulation of a digital community currency
Source: Sci Rep. 2023 Apr 11;13:5864. doi: 10.1038/s41598-023-33184-1 (PMC10088680; doi:10.1038/s41598-023-33184-1)
Supplement: Supplementary file 4 — Supplementary Information 4. [file 41598_2023_33184_MOESM4_ESM.pdf]

````
---
title: "Empirical"
author: "Teodoro Criscione"
date: "2023-02"
---
# Loading Packages
```{r}
library(igraph)
library(survival)
library(network)
library(sna)
library(intergraph)
library(parallel)
detectCores()
```

```{r}
setwd("/Users/teodorocriscione/Desktop/Correction_Circulation_Paper/")
```

# Functions
1. createGraph
```{r}
createGraph <- function(module, submodule){
  address = './empirical_submodules_networks/'
  submodule_file = paste(address,'sarafu_reg_users_', module, '_', submodule, '.net')
  submodule_file = gsub(" ","", paste(submodule_file))
  gg <- igraph::read.graph(submodule_file, format = "pajek")
  return(gg)}
```

2. countCycles
```{r}
countCycles <- function(g){
  g.adj <- get.adjacency(g, sparse = FALSE)
  kcycles.g <- sna::kcycle.census(g.adj, maxlen = 5, mode = "digraph",
                                     tabulate.by.vertex = TRUE, cycle.comembership =
c("none"))
  number_cycles <- c('\n',
                     as.numeric(kcycles.g[[1]][[1]]),
                     as.numeric(kcycles.g[[1]][[2]]),
                     as.numeric(kcycles.g[[1]][[3]]),
                     as.numeric(kcycles.g[[1]][[4]]))
  rm(kcycles.g)
  return(number_cycles)}
```

3. writeMe
```{r}
writeMe <- function(module, submodule, output, string_output){
  g <- createGraph(module, submodule)
  string_to_print <-  append(output, c(vcount(g), ecount(g), module, submodule))
  address = './'
  file_name <- gsub(" ", "", paste(address, string_output,".txt"))
  cat(string_to_print, file=file_name, append=T, sep=',')
}
```

# Apply

```{r}
submodules <- cbind(c(1, 1), c(1, 2), c(1, 3), c(1, 4), c(1, 5), c(1, 6),
              c(1, 7), c(1, 8), c(1, 9), c(1, 10), c(1, 13),
              c(2, 1), c(2, 2), c(2, 3), c(2, 4), c(2, 5), c(2, 6),
              c(2, 7), c(2, 8), c(2, 9), c(2, 10), c(2, 11),
              c(2, 12), c(2, 13), c(2, 14), c(2, 15),
              c(3, 1), c(3, 2), c(3, 3), c(3, 4), c(3, 6),
              c(4, 1), c(4, 2), c(4, 3), c(4, 4),
              c(5, 1), c(5, 2))
````
````

~~~
dim(submodules)[2]
```

```{r}
outputFunction <- function(i){
  module = submodules[,i][1]
  submodule = submodules[,i][2]
  g = createGraph(module, submodule)
  writeMe(module, submodule, output=countCycles(g), string_output='empirical_cycles')
  }
```

```{r}
system.time(save <- lapply(1:dim(submodules)[2], outputFunction))
```
~~~

CYCLE COUNTS -- EMPIRICAL NETWORKS

,4358,72275,2150260,67199815,1379,24816,1,1
,1215,5533,44988,415560,567,3051,1,2
,2302,28075,761364,21872662,1522,7563,1,3
,1757,7675,95011,1262914,2002,6236,1,4
,1501,11952,167902,2524173,1955,5392,1,5
,410,1989,20417,214622,238,1087,1,6
,636,4491,61188,908521,182,1401,1,7
,609,3704,47796,690237,344,1695,1,8
,442,2354,19239,179311,168,1166,1,9
,368,1292,9475,76313,229,930,1,10
,129,3,1,0,210,343,1,13
,4048,7334,72248,770418,4286,13074,2,1
,2248,8044,113246,1788572,3226,10461,2,2
,1361,2332,13764,98365,3147,7223,2,3
,992,1317,7355,48701,1967,4613,2,4
,740,1082,6572,42696,1634,3947,2,5
,687,1447,10361,70461,640,2214,2,6
,366,855,4873,26750,888,2061,2,7
,447,871,5147,31240,1199,2749,2,8
,362,484,1887,7814,623,1415,2,9
,183,319,1342,5897,374,840,2,10
,226,263,1243,4962,500,1166,2,11
,66,141,514,1948,297,559,2,12
,72,116,326,833,181,345,2,13
,159,530,3065,18424,218,556,2,14
,229,1042,9601,88123,165,773,2,15
,721,3508,28674,252245,937,2878,3,1
,889,3107,25321,225412,537,2427,3,2
,542,2506,20656,184645,444,1693,3,3
,752,643,3024,16314,1225,2498,3,4
,161,174,546,1624,222,459,3,6
,3324,3843,15752,78731,3417,9773,4,1
,2029,1477,6049,32197,2108,5542,4,2
,133,44,63,93,180,379,4,3
,68,50,76,103,136,273,4,4
,86,50,72,122,276,398,5,1
,5,0,0,0,168,175,5,2

````
---
title: "ER"
author: "Teodoro Criscione"
date: "2023-02"
---

# Loading Packages
```{r}
library(igraph)
library(survival)
library(network)
library(sna)
library(intergraph)
library(parallel)
detectCores()
```

```{r}
setwd("/Users/teodorocriscione/Desktop/Correction_Circulation_Paper/")
```

# Functions
1. createGraph
```{r}
createGraph <- function(module, submodule){
  address = './empirical_submodules_networks/'
  submodule_file = paste(address,'sarafu_reg_users_', module, '_', submodule, '.net')
  submodule_file = gsub(" ","", paste(submodule_file))
  gg <- igraph::read.graph(submodule_file, format = "pajek")
  return(gg)}
```

2. createERmodel
```{r}
createERmodel <- function(g){
  ER <- erdos.renyi.game( n=vcount(g), p.or.m=ecount(g), type = c("gnm"), directed =
TRUE, loops = FALSE)
  return(ER)}
```

3. countCycles
```{r}
countCycles <- function(g){
  g.adj <- get.adjacency(g, sparse = FALSE)
  kcycles.g <- sna::kcycle.census(g.adj, maxlen = 5, mode = "digraph",
                                  tabulate.by.vertex = TRUE, cycle.comembership =
c("none"))
  number_cycles <- c('\n',
                     as.numeric(kcycles.g[[1]][[1]]),
                     as.numeric(kcycles.g[[1]][[2]]),
                     as.numeric(kcycles.g[[1]][[3]]),
                     as.numeric(kcycles.g[[1]][[4]]))
  rm(kcycles.g)
  return(number_cycles)}
```

3. writeMe
```{r}
writeMe <- function(module, submodule, model, output, string_output){
  g <- createGraph(module, submodule)
  string_to_print <-  append(output, c(vcount(g), ecount(g), module, submodule, model))
  address = './'
  file_name <- gsub(" ", "", paste(address, string_output,".txt"))
  cat(string_to_print, file=file_name, append=T, sep=',')
}
```

# Implementation
````

````
```{r}
submodules <- cbind(c(1, 1), c(1, 2), c(1, 3), c(1, 4), c(1, 5), c(1, 6),
              c(1, 7), c(1, 8), c(1, 9), c(1, 10), c(1, 13),
              c(2, 1), c(2, 2), c(2, 3), c(2, 4), c(2, 5), c(2, 6),
              c(2, 7), c(2, 8), c(2, 9), c(2, 10), c(2, 11),
              c(2, 12), c(2, 13), c(2, 14), c(2, 15),
              c(3, 1), c(3, 2), c(3, 3), c(3, 4), c(3, 6),
              c(4, 1), c(4, 2), c(4, 3), c(4, 4),
              c(5, 1), c(5, 2))
dim(submodules)[2]
```

```{r}
output_f <- function(i){
module <- submodules[,i][1]
submodule <- submodules[,i][2]
g <- createGraph(module, submodule)
for (i in 0:99){
er <- createERmodel(g)
writeMe(module, submodule, model=i, output=countCycles(er), string_output='ER_cycles')
}
}
```

```{r}
system.time(save <- mclapply(1:dim(submodules)[2], output_f, mc.cores = 6))
```
````

CYCLE COUNTS -- ER NETWORKS

```
,14,32,95,364,238,1087,1,6,0
,11,36,101,389,238,1087,1,6,1
,11,38,120,456,238,1087,1,6,2
,8,36,114,364,238,1087,1,6,3
,8,30,103,403,238,1087,1,6,4
,9,37,129,364,238,1087,1,6,5
,9,45,102,352,238,1087,1,6,6
,12,30,100,343,238,1087,1,6,7
,16,32,97,371,238,1087,1,6,8
,16,37,104,350,238,1087,1,6,9
,14,43,218,866,567,3051,1,2,0
,11,30,101,401,238,1087,1,6,10
,9,35,97,455,238,1087,1,6,11
,12,32,93,378,238,1087,1,6,12
,17,31,95,375,238,1087,1,6,13
,9,35,117,437,238,1087,1,6,14
,13,23,112,366,238,1087,1,6,15
,13,34,104,318,238,1087,1,6,16
,9,17,87,377,238,1087,1,6,17
,10,22,103,363,238,1087,1,6,18
,7,35,115,391,238,1087,1,6,19
,14,29,95,393,238,1087,1,6,20
,17,43,191,907,567,3051,1,2,1
,13,44,97,445,238,1087,1,6,21
,11,41,118,456,238,1087,1,6,22
,10,28,106,427,238,1087,1,6,23
,13,43,116,404,238,1087,1,6,24
,14,23,79,333,238,1087,1,6,25
,10,25,95,426,238,1087,1,6,26
,10,34,112,405,238,1087,1,6,27
,2,7,12,44,1955,5392,1,5,0
,13,43,94,355,238,1087,1,6,28
,8,35,109,392,238,1087,1,6,29
,16,36,98,381,238,1087,1,6,30
,10,30,126,399,238,1087,1,6,31
,6,33,106,409,238,1087,1,6,32
,16,44,201,832,567,3051,1,2,2
,14,36,93,389,238,1087,1,6,33
,11,32,102,389,238,1087,1,6,34
,6,22,95,357,238,1087,1,6,35
,7,31,115,385,238,1087,1,6,36
,5,9,22,55,2002,6236,1,4,0
,5,28,78,340,238,1087,1,6,37
,9,37,115,379,238,1087,1,6,38
,10,30,137,410,238,1087,1,6,39
,13,27,106,350,238,1087,1,6,40
,10,37,109,362,238,1087,1,6,41
,14,32,96,367,238,1087,1,6,42
,11,43,177,945,567,3051,1,2,3
,6,29,100,434,238,1087,1,6,43
,11,30,113,422,238,1087,1,6,44
,4,25,103,361,238,1087,1,6,45
```

,12,37,97,362,238,1087,1,6,46
,14,39,114,420,238,1087,1,6,47
,12,32,121,442,238,1087,1,6,48
,9,29,101,333,238,1087,1,6,49
,4,10,14,44,1955,5392,1,5,1
,8,26,99,368,238,1087,1,6,50
,10,38,112,406,238,1087,1,6,51
,13,32,110,422,238,1087,1,6,52
,14,54,217,856,567,3051,1,2,4
,11,31,98,358,238,1087,1,6,53
,5,40,122,384,238,1087,1,6,54
,12,36,106,384,238,1087,1,6,55
,6,31,141,401,238,1087,1,6,56
,9,28,99,381,238,1087,1,6,57
,9,38,111,355,238,1087,1,6,58
,17,34,100,437,238,1087,1,6,59
,11,27,109,405,238,1087,1,6,60
,12,31,94,380,238,1087,1,6,61
,14,36,116,468,238,1087,1,6,62
,7,30,114,349,238,1087,1,6,63
,11,38,93,386,238,1087,1,6,64
,16,49,200,963,567,3051,1,2,5
,13,32,92,350,238,1087,1,6,65
,15,31,92,380,238,1087,1,6,66
,11,28,109,393,238,1087,1,6,67
,12,33,91,345,238,1087,1,6,68
,16,23,92,414,238,1087,1,6,69
,5,33,98,335,238,1087,1,6,70
,3,13,17,67,2002,6236,1,4,1
,7,42,98,415,238,1087,1,6,71
,11,33,152,588,1522,7563,1,3,0
,9,28,123,376,238,1087,1,6,72
,13,34,91,349,238,1087,1,6,73
,2,8,12,30,1955,5392,1,5,2
,12,35,113,411,238,1087,1,6,74
,11,33,132,487,238,1087,1,6,75
,16,34,107,405,238,1087,1,6,76
,22,49,198,947,567,3051,1,2,6
,10,39,120,351,238,1087,1,6,77
,7,34,102,387,238,1087,1,6,78
,13,23,109,437,238,1087,1,6,79
,8,24,109,361,238,1087,1,6,80
,13,30,107,382,238,1087,1,6,81
,13,33,96,325,238,1087,1,6,82
,7,36,104,433,238,1087,1,6,83
,11,24,110,387,238,1087,1,6,84
,9,31,130,427,238,1087,1,6,85
,11,28,97,361,238,1087,1,6,86
,16,28,100,382,238,1087,1,6,87
,10,34,102,349,238,1087,1,6,88
,18,53,193,890,567,3051,1,2,7
,9,24,103,413,238,1087,1,6,89
,11,24,109,436,238,1087,1,6,90
,9,26,95,364,238,1087,1,6,91

,9,29,127,404,238,1087,1,6,92
,14,34,112,362,238,1087,1,6,93
,14,32,90,353,238,1087,1,6,94
,9,28,116,388,238,1087,1,6,95
,5,9,11,26,1955,5392,1,5,3
,15,36,100,320,238,1087,1,6,96
,14,17,114,345,238,1087,1,6,97
,21,35,101,356,238,1087,1,6,98
,14,51,206,914,567,3051,1,2,8
,8,37,117,433,238,1087,1,6,99
,4,14,16,50,2002,6236,1,4,2
,25,43,215,827,567,3051,1,2,9
,3,8,15,28,1955,5392,1,5,4
,10,51,218,958,567,3051,1,2,10
,17,57,180,909,567,3051,1,2,11
,11,12,21,60,2002,6236,1,4,3
,6,6,13,29,1955,5392,1,5,5
,14,61,198,959,567,3051,1,2,12
,16,42,160,566,1522,7563,1,3,1
,16,49,222,959,567,3051,1,2,13
,5,10,15,25,1955,5392,1,5,6
,7,50,194,881,567,3051,1,2,14
,2,12,18,55,2002,6236,1,4,4
,18,51,193,818,567,3051,1,2,15
,1,6,14,45,1955,5392,1,5,7
,19,65,202,861,567,3051,1,2,16
,6,59,232,894,567,3051,1,2,17
,4,10,26,42,2002,6236,1,4,5
,5,6,8,28,1955,5392,1,5,8
,16,49,220,893,567,3051,1,2,18
,19,46,204,949,567,3051,1,2,19
,7,25,171,594,1522,7563,1,3,2
,9,56,207,931,567,3051,1,2,20
,4,8,12,31,1955,5392,1,5,9
,3,6,16,38,2002,6236,1,4,6
,14,56,246,913,567,3051,1,2,21
,12,53,210,844,567,3051,1,2,22
,4,9,13,31,1955,5392,1,5,10
,4,9,21,57,4286,13074,2,1,0
,14,51,191,917,567,3051,1,2,23
,3,9,27,54,2002,6236,1,4,7
,8,42,208,855,567,3051,1,2,24
,7,7,24,31,1955,5392,1,5,11
,8,37,204,906,567,3051,1,2,25
,15,33,167,581,1522,7563,1,3,3
,17,42,211,911,567,3051,1,2,26
,7,6,17,62,2002,6236,1,4,8
,2,9,25,34,1955,5392,1,5,12
,8,52,204,824,567,3051,1,2,27
,17,46,198,923,567,3051,1,2,28
,2,7,13,26,1955,5392,1,5,13
,13,61,233,980,567,3051,1,2,29
,5,16,16,59,2002,6236,1,4,9
,19,51,210,917,567,3051,1,2,30

This notebook is based on NEMtropy Github repository.

In [1]:
```
%pwd
```

Out[1]:
```
'/Users/teodorocriscione/Dropbox/My Mac (Teodoros-MacBook-Pro.local)/Deskto
p/Correction_Circulation_Paper'
```

In [2]:
```
import os
import numpy as np
import networkx as nx
import pandas as pd
from NEMtropy import DirectedGraph
from NEMtropy import matrix_generator as mg
from NEMtropy.network_functions import build_adjacency_from_edgelist
```

In [3]:
```
import warnings
warnings.filterwarnings("ignore")
```

In [4]:
```
# Define directories
homedir = os.path.expanduser("./")
datadir = os.path.join(homedir,'empirical_submodules_networks')
resultsdir = os.path.join(homedir,'ERGs_all_graphs')
try:
    os.mkdir(resultsdir)
except FileExistsError:
    pass
```

In [5]:
```
submodules = [(1, 1), (1, 2), (1, 3), (1, 4), (1, 5), (1, 6),
              (1, 7), (1, 8), (1, 9), (1, 10), (1, 13),
              (2, 1), (2, 2), (2, 3), (2, 4), (2, 5), (2, 6),
              (2, 7), (2, 8), (2, 9), (2, 10), (2, 11),
              (2, 12), (2, 13), (2, 14), (2, 15),
              (3, 1), (3, 2), (3, 3), (3, 4), (3, 6),
              (4, 1), (4, 2), (4, 3), (4, 4),
              (5, 1), (5, 2)]
```

In [6]:
```
def convert_to_DiGraph(network):
    return nx.DiGraph(list(set(network.edges())))
```

In [7]:
```
def createGraph(module_n, submodule_n):
    name_file = datadir+"/sarafu_reg_users_"+str(module_n)+"_"+str(submodule
    G = nx.read_pajek(name_file)
    return convert_to_DiGraph(G)
```

In [8]:
```
def createDCM_ERGs(sample_size, module_n, submodule_n):
    mainfolder = resultsdir
    # adjacency
    network = createGraph(module_n, submodule_n)
    print("For module {} and submodule {} = ".format(module_n, submodule_n),
    adj_digraph = nx.to_numpy_array(network,weight=None)
    # initialize using adjacency
    graph = DirectedGraph(adj_digraph)
    # train the model to create null models having (on average) the same deg
    graph.solve_tool(model="dcm_exp",
                     method="newton",
                     initial_guess="random")
    print("For module {} and submodule {}. The largest error on the degree s
    # sample_size random copies of the graph saved as edgelist.
    graph.ensemble_sampler(sample_size,
                           cpu_n=6,
                           output_dir=mainfolder+"/submodule_"+str(module_n)
    return
```

DirectedGraph instance is initialised using adjacency matrix.

Generate 30 random versions of our directed network having (on average) the same degree sequence of the orginal one. These can be used to compare same properties of the original network with their expected values under a random null model preserving the degree sequence.

`solve_tool` function solves, for the underlying graph instance, the selected "model" using "method" and "initial_guess" as starting point of the optimization.

In [9]:
```
counter = 0
for s in submodules:
    createDCM_ERGs(sample_size=100, module_n=s[0], submodule_n=s[1])
    counter += 1
    print(counter)
    print()
```

```
For module 1 and submodule 1 =  DiGraph with 1379 nodes and 24816 edges

solution error = 2.1301183039668103e-10
For module 1 and submodule 1. The largest error on the degree sequence is
2.1301183039668103e-10
1

For module 1 and submodule 2 =  DiGraph with 567 nodes and 3051 edges

solution error = 1.237229752870661e-06
For module 1 and submodule 2. The largest error on the degree sequence is
1.237229752870661e-06
2

For module 1 and submodule 3 =  DiGraph with 1522 nodes and 7563 edges

solution error = 8.478280663526405e-06
For module 1 and submodule 3. The largest error on the degree sequence is
8.478280663526405e-06
3

For module 1 and submodule 4 =  DiGraph with 2002 nodes and 6236 edges

solution error = 2.93354540659152e-09
For module 1 and submodule 4. The largest error on the degree sequence is
2.93354540659152e-09
4

For module 1 and submodule 5 =  DiGraph with 1955 nodes and 5392 edges

solution error = 3.705185047664372e-10
For module 1 and submodule 5. The largest error on the degree sequence is
3.705185047664372e-10
5

For module 1 and submodule 6 =  DiGraph with 238 nodes and 1087 edges

solution error = 7.87436893467941e-09
For module 1 and submodule 6. The largest error on the degree sequence is
7.87436893467941e-09
6

For module 1 and submodule 7 =  DiGraph with 182 nodes and 1401 edges

solution error = 7.54839986960576e-07
For module 1 and submodule 7. The largest error on the degree sequence is
7.54839986960576e-07
7

For module 1 and submodule 8 =  DiGraph with 344 nodes and 1695 edges

solution error = 8.165566001139268e-10
For module 1 and submodule 8. The largest error on the degree sequence is
8.165566001139268e-10
8

For module 1 and submodule 9 =  DiGraph with 168 nodes and 1166 edges

solution error = 2.28048548933657e-08
```

```
For module 1 and submodule 9. The largest error on the degree sequence is
2.28048548933657e-08
9

For module 1 and submodule 10 =  DiGraph with 229 nodes and 930 edges

solution error = 9.237055564881302e-14
For module 1 and submodule 10. The largest error on the degree sequence is
9.237055564881302e-14
10

For module 1 and submodule 13 =  DiGraph with 210 nodes and 343 edges

solution error = 5.966116489730666e-11
For module 1 and submodule 13. The largest error on the degree sequence is
5.966116489730666e-11
11

For module 2 and submodule 1 =  DiGraph with 4286 nodes and 13074 edges

solution error = 2.3273549754065925e-09
For module 2 and submodule 1. The largest error on the degree sequence is
2.3273549754065925e-09
12

For module 2 and submodule 2 =  DiGraph with 3226 nodes and 10461 edges

solution error = 3.8129768338279746e-06
For module 2 and submodule 2. The largest error on the degree sequence is
3.8129768338279746e-06
13

For module 2 and submodule 3 =  DiGraph with 3147 nodes and 7223 edges

solution error = 5.552546400444669e-08
For module 2 and submodule 3. The largest error on the degree sequence is
5.552546400444669e-08
14

For module 2 and submodule 4 =  DiGraph with 1967 nodes and 4613 edges

solution error = 4.30368984893903e-09
For module 2 and submodule 4. The largest error on the degree sequence is
4.30368984893903e-09
15

For module 2 and submodule 5 =  DiGraph with 1634 nodes and 3947 edges

solution error = 1.998131438085693e-09
For module 2 and submodule 5. The largest error on the degree sequence is
1.998131438085693e-09
16

For module 2 and submodule 6 =  DiGraph with 640 nodes and 2214 edges

solution error = 1.0231815394945443e-12
For module 2 and submodule 6. The largest error on the degree sequence is
1.0231815394945443e-12
17
```

```
For module 2 and submodule 7 =  DiGraph with 888 nodes and 2061 edges

solution error = 1.2402239235598245e-09
For module 2 and submodule 7. The largest error on the degree sequence is
1.2402239235598245e-09
18

For module 2 and submodule 8 =  DiGraph with 1199 nodes and 2749 edges

solution error = 2.690092593127247e-10
For module 2 and submodule 8. The largest error on the degree sequence is
2.690092593127247e-10
19

For module 2 and submodule 9 =  DiGraph with 623 nodes and 1415 edges

solution error = 1.7139853980552289e-09
For module 2 and submodule 9. The largest error on the degree sequence is
1.7139853980552289e-09
20

For module 2 and submodule 10 =  DiGraph with 374 nodes and 840 edges

solution error = 1.1767305352350377e-08
For module 2 and submodule 10. The largest error on the degree sequence is
1.1767305352350377e-08
21

For module 2 and submodule 11 =  DiGraph with 500 nodes and 1166 edges

solution error = 6.821210263296962e-13
For module 2 and submodule 11. The largest error on the degree sequence is
6.821210263296962e-13
22

For module 2 and submodule 12 =  DiGraph with 297 nodes and 559 edges

solution error = 4.374234308102132e-11
For module 2 and submodule 12. The largest error on the degree sequence is
4.374234308102132e-11
23

For module 2 and submodule 13 =  DiGraph with 181 nodes and 345 edges

solution error = 4.2086334417490434e-12
For module 2 and submodule 13. The largest error on the degree sequence is
4.2086334417490434e-12
24

For module 2 and submodule 14 =  DiGraph with 218 nodes and 556 edges

solution error = 4.42299086245157e-10
For module 2 and submodule 14. The largest error on the degree sequence is
4.42299086245157e-10
25

For module 2 and submodule 15 =  DiGraph with 165 nodes and 773 edges
```

```
solution error = 1.1126966015240214e-09
For module 2 and submodule 15. The largest error on the degree sequence is
1.1126966015240214e-09
26

For module 3 and submodule 1 =  DiGraph with 937 nodes and 2878 edges

solution error = 1.6025033744426764e-08
For module 3 and submodule 1. The largest error on the degree sequence is
1.6025033744426764e-08
27

For module 3 and submodule 2 =  DiGraph with 537 nodes and 2427 edges

solution error = 4.25472990173148e-11
For module 3 and submodule 2. The largest error on the degree sequence is
4.25472990173148e-11
28

For module 3 and submodule 3 =  DiGraph with 444 nodes and 1693 edges

solution error = 9.3551058011343e-09
For module 3 and submodule 3. The largest error on the degree sequence is
9.3551058011343e-09
29

For module 3 and submodule 4 =  DiGraph with 1225 nodes and 2498 edges

solution error = 2.3874235921539366e-12
For module 3 and submodule 4. The largest error on the degree sequence is
2.3874235921539366e-12
30

For module 3 and submodule 6 =  DiGraph with 222 nodes and 459 edges

solution error = 1.906378055416269e-08
For module 3 and submodule 6. The largest error on the degree sequence is
1.906378055416269e-08
31

For module 4 and submodule 1 =  DiGraph with 3417 nodes and 9773 edges

solution error = 3.830229656998085e-06
For module 4 and submodule 1. The largest error on the degree sequence is
3.830229656998085e-06
32

For module 4 and submodule 2 =  DiGraph with 2108 nodes and 5542 edges

solution error = 4.810751796924251e-11
For module 4 and submodule 2. The largest error on the degree sequence is
4.810751796924251e-11
33

For module 4 and submodule 3 =  DiGraph with 180 nodes and 379 edges

solution error = 5.4461102294567354e-11
For module 4 and submodule 3. The largest error on the degree sequence is
5.4461102294567354e-11
```

```
34

For module 4 and submodule 4 =  DiGraph with 136 nodes and 273 edges

solution error = 7.481570918344005e-11
For module 4 and submodule 4. The largest error on the degree sequence is
7.481570918344005e-11
35

For module 5 and submodule 1 =  DiGraph with 276 nodes and 398 edges

solution error = 1.0124143745571246e-09
For module 5 and submodule 1. The largest error on the degree sequence is
1.0124143745571246e-09
36

For module 5 and submodule 2 =  DiGraph with 168 nodes and 175 edges

solution error = 9.947598300641403e-14
For module 5 and submodule 2. The largest error on the degree sequence is
9.947598300641403e-14
37
```

In [ ]:

````
---
title: "ERGs_Analysis"
author: "Teodoro Criscione"
date: "2023-02"
---
ERGs configuration models. This notebook is only for the analysis.

# Loading Packages
```{r}
library(igraph)
library(survival)
library(network)
library(sna)
library(intergraph)
library(parallel)
detectCores()
```

```{r}
setwd("/Users/teodorocriscione/Desktop/Correction_Circulation_Paper/")
```

# Functions
1. createGraph
```{r}
createGraph <- function(module_n, submodule_n, model_number){
  address = "./ERGs_all_graphs/"
  vector_strings = paste(address, "submodule_",
                         toString(module_n),"_",
                         toString(submodule_n), "/",
                         toString(model_number), ".txt")
  file_name_graph = gsub(" ","",paste(vector_strings))
  dd <- read.table(file_name_graph)
  gg <- igraph::graph_from_data_frame(dd, directed=T)
  return(gg)}
```

2. countCycles
```{r}
countCycles <- function(g){
  g.adj <- get.adjacency(g, sparse = FALSE)
  kcycles.g <- sna::kcycle.census(g.adj, maxlen = 5, mode = "digraph",
                                     tabulate.by.vertex = TRUE, cycle.comembership =
c("none"))
  number_cycles <- c('\n',
                     as.numeric(kcycles.g[[1]][[1]]),
                     as.numeric(kcycles.g[[1]][[2]]),
                     as.numeric(kcycles.g[[1]][[3]]),
                     as.numeric(kcycles.g[[1]][[4]]))
  rm(kcycles.g)
  return(number_cycles)}
```

3. writeMe
```{r}
writeMe <- function(module, submodule, model, output, string_output){
  g <- createGraph(module, submodule, model)
  string_to_print <-  append(output, c(vcount(g), ecount(g), module, submodule, model))
  address = './'
  file_name <- gsub(" ", "", paste(address, string_output,".txt"))
  cat(string_to_print, file=file_name, append=T, sep=',')
}
```

# Implementation

```{r}
submodules <- cbind(c(1, 1), c(1, 2), c(1, 3), c(1, 4), c(1, 5), c(1, 6),
              c(1, 7), c(1, 8), c(1, 9), c(1, 10), c(1, 13),
````
````

````
              c(2, 1), c(2, 2), c(2, 3), c(2, 4), c(2, 5), c(2, 6),
              c(2, 7), c(2, 8), c(2, 9), c(2, 10), c(2, 11),
              c(2, 12), c(2, 13), c(2, 14), c(2, 15),
              c(3, 1), c(3, 2), c(3, 3), c(3, 4), c(3, 6),
              c(4, 1), c(4, 2), c(4, 3), c(4, 4),
              c(5, 1), c(5, 2))
dim(submodules)[2]
```

```{r}
output_f <- function(i){
module <- submodules[,i][1]
submodule <- submodules[,i][2]
for (i in 0:99){
gg <- createGraph(module, submodule, model_number=i)
writeMe(module, submodule, model=i, output=countCycles(gg), string_output='ERGs_cycles')
}}
```

```{r}
system.time(save <- mclapply(1:dim(submodules)[2], output_f, mc.cores = 6))
```
````
````

CYCLE COUNTS -- RD NETWORKS

```
,141,1199,13206,141980,210,1075,1,6,0
,131,1259,13373,144554,205,1091,1,6,1
,124,1092,11084,114672,205,1026,1,6,2
,146,1465,16728,193382,207,1109,1,6,3
,145,1255,13629,146297,204,1088,1,6,4
,141,1348,15024,169159,210,1075,1,6,5
,150,1218,13051,140751,218,1099,1,6,6
,134,1290,14376,158342,205,1043,1,6,7
,148,1404,15373,172221,211,1099,1,6,8
,107,1087,11516,129215,497,3052,1,2,0
,144,1425,16271,185052,208,1091,1,6,9
,134,1176,12642,134163,208,1081,1,6,10
,138,1208,13043,140701,212,1082,1,6,11
,134,1352,15420,173382,208,1079,1,6,12
,146,1370,15060,167959,205,1069,1,6,13
,140,1276,13875,150895,215,1095,1,6,14
,121,1223,13088,141354,207,1050,1,6,15
,149,1357,15507,173599,218,1099,1,6,16
,133,1248,13151,143142,210,1086,1,6,17
,147,1531,17822,206338,211,1123,1,6,18
,114,1077,11241,126802,505,3056,1,2,1
,148,1286,13912,151595,209,1067,1,6,19
,134,1206,13111,140456,205,1053,1,6,20
,154,1466,16619,190550,200,1109,1,6,21
,129,1096,11465,118457,213,1051,1,6,22
,120,1095,11310,117046,202,1013,1,6,23
,134,1241,13567,145979,215,1086,1,6,24
,140,1389,15134,170318,214,1105,1,6,25
,144,1305,14537,162378,211,1128,1,6,26
,101,955,9821,105424,512,3019,1,2,2
,157,1445,16308,184745,211,1112,1,6,27
,151,1479,17205,198057,206,1096,1,6,28
,149,1355,15247,169924,205,1065,1,6,29
,128,1293,13827,152157,219,1092,1,6,30
,150,1376,15687,178251,206,1099,1,6,31
,130,1131,12120,127710,209,1040,1,6,32
,166,1788,22553,302782,1457,5378,1,5,0
,111,1025,10456,106793,214,1069,1,6,33
,141,1298,14020,155757,212,1097,1,6,34
,95,1040,10565,116144,487,3074,1,2,3
,136,1328,14525,160001,217,1089,1,6,35
,147,1495,16727,191885,206,1093,1,6,36
,134,1160,12142,127329,210,1020,1,6,37
,126,1252,13198,142456,209,1108,1,6,38
,140,1370,15342,172950,209,1126,1,6,39
,138,1149,12504,133427,204,1053,1,6,40
,130,1258,13399,144873,206,1057,1,6,41
,134,1289,14185,156543,213,1077,1,6,42
,144,1244,13097,140526,210,1054,1,6,43
,109,941,9453,100927,496,2995,1,2,4
,146,1359,15024,167945,206,1058,1,6,44
,141,1288,14365,157752,200,1078,1,6,45
```

,165,1439,16910,194684,204,1130,1,6,46
,174,2072,27767,375706,1591,6144,1,4,0
,141,1308,14538,161745,205,1071,1,6,47
,135,1328,14644,161609,204,1055,1,6,48
,128,1306,14378,158311,210,1051,1,6,49
,149,1340,15253,171678,217,1091,1,6,50
,136,1255,13760,149588,215,1066,1,6,51
,129,1179,12338,131507,209,1072,1,6,52
,99,1059,11107,124535,496,3068,1,2,5
,143,1227,13222,141811,211,1076,1,6,53
,132,1311,14368,158502,207,1054,1,6,54
,141,1272,13973,150752,207,1077,1,6,55
,135,1175,12932,140668,212,1098,1,6,56
,147,1445,16133,183636,213,1111,1,6,57
,143,1304,14661,163153,206,1069,1,6,58
,153,1246,13607,147409,200,1060,1,6,59
,142,1340,14748,163365,214,1070,1,6,60
,138,1324,14481,161224,220,1080,1,6,61
,106,1043,11269,124956,504,3102,1,2,6
,135,1292,14185,155519,202,1115,1,6,62
,150,1350,14638,162326,207,1090,1,6,63
,137,1607,20175,262004,1414,5298,1,5,1
,146,1299,14125,154379,211,1078,1,6,64
,125,1226,12898,137609,208,1051,1,6,65
,131,1239,13008,140315,207,1060,1,6,66
,148,1375,15371,174031,204,1097,1,6,67
,150,1386,15614,175610,207,1100,1,6,68
,134,1237,12935,138760,205,1058,1,6,69
,104,925,9714,103998,504,3060,1,2,7
,158,1400,16169,184549,208,1136,1,6,70
,123,1212,12984,139760,214,1067,1,6,71
,140,1322,14331,157341,207,1067,1,6,72
,141,1383,15709,178083,210,1120,1,6,73
,131,1156,12552,134917,213,1071,1,6,74
,124,1129,12165,128383,216,1083,1,6,75
,145,1382,15771,177053,212,1075,1,6,76
,136,1221,13056,141409,201,1054,1,6,77
,145,1349,14881,165475,212,1102,1,6,78
,104,1037,11148,125135,491,3021,1,2,8
,142,1312,14891,164898,218,1151,1,6,79
,157,1421,16397,187812,211,1103,1,6,80
,152,1242,13288,144291,203,1045,1,6,81
,141,1293,14507,161230,213,1087,1,6,82
,142,1386,16000,182318,207,1112,1,6,83
,135,1243,13719,150207,212,1103,1,6,84
,126,1219,13219,142925,200,1058,1,6,85
,142,1369,15434,174252,213,1087,1,6,86
,106,1008,10500,114966,508,3072,1,2,9
,147,1577,17845,210069,211,1087,1,6,87
,141,1257,13400,146123,205,1079,1,6,88
,165,1483,17236,200128,203,1125,1,6,89
,137,1303,14863,163848,201,1075,1,6,90
,111,1025,10456,106793,214,1069,1,6,91
,144,1300,14521,163307,208,1097,1,6,92

,153,1677,21153,276027,1456,5384,1,5,2
,145,1445,16457,188274,205,1095,1,6,93
,117,1170,12332,131519,207,1065,1,6,94
,195,2536,35040,513854,1585,6141,1,4,1
,121,1203,12976,139803,197,1039,1,6,95
,101,1001,10520,116940,505,3080,1,2,10
,129,1309,14360,159019,203,1074,1,6,96
,153,1360,15586,174153,202,1084,1,6,97
,151,1425,16505,187775,215,1093,1,6,98
,124,1160,12578,134817,217,1088,1,6,99
,107,985,10081,109715,505,3037,1,2,11
,93,883,8768,91222,505,2992,1,2,12
,104,1005,10589,116900,506,3028,1,2,13
,149,1726,21447,279635,1439,5305,1,5,3
,109,986,10197,110238,500,3046,1,2,14
,91,856,9141,97563,502,2946,1,2,15
,110,1026,10724,120097,493,3037,1,2,16
,203,2572,35574,519739,1593,6188,1,4,2
,119,1061,11237,125544,504,3072,1,2,17
,154,1592,19996,256188,1441,5396,1,5,4
,83,924,9722,102007,495,3002,1,2,18
,104,965,9721,105089,506,2992,1,2,19
,481,9319,211551,5002835,1169,7616,1,3,0
,103,869,8718,90944,499,3029,1,2,20
,111,956,9883,107839,498,3022,1,2,21
,146,1693,21235,277858,1488,5403,1,5,5
,174,1974,26145,357505,1583,6120,1,4,3
,102,1009,11329,125231,504,3075,1,2,22
,109,964,9956,106537,492,2951,1,2,23
,85,824,8029,82539,501,2943,1,2,24
,155,1590,19249,249045,1464,5402,1,5,6
,105,1005,10322,112047,498,3004,1,2,25
,114,1079,10926,121007,502,3069,1,2,26
,89,901,9297,97648,500,3006,1,2,27
,197,2219,30237,421796,1616,6204,1,4,4
,103,1018,10666,118785,503,3034,1,2,28
,149,1717,21169,278882,1463,5450,1,5,7
,124,990,9640,104152,503,3012,1,2,29
,79,886,8932,93457,511,2987,1,2,30
,91,860,9081,96113,503,3036,1,2,31
,98,973,10158,110898,497,3053,1,2,32
,160,1706,21521,284492,1487,5388,1,5,8
,99,899,8702,90234,493,2968,1,2,33
,198,2268,32095,455827,1592,6217,1,4,5
,111,1031,10711,118324,503,3092,1,2,34
,112,958,9700,103865,509,2959,1,2,35
,135,1716,21115,279195,1455,5470,1,5,9
,106,981,10440,114067,503,3077,1,2,36
,102,886,9198,98191,502,3040,1,2,37
,455,8363,180161,4083017,1170,7319,1,3,1
,105,970,10249,112617,503,3055,1,2,38
,154,1471,17854,223773,1451,5263,1,5,10
,92,947,9783,105964,503,3026,1,2,39
,186,2305,31075,441147,1572,6301,1,4,6
