# Supplementary material for: Circulation of a digital community currency
Source: Sci Rep. 2023 Apr 11;13:5864. doi: 10.1038/s41598-023-33184-1 (PMC10088680; doi:10.1038/s41598-023-33184-1)
Supplement: Supplementary file 5 — Supplementary Information 5. [file 41598_2023_33184_MOESM5_ESM.html]

SI\_5


In [1]:

```
%pwd
```

Out[1]:

```
'/Users/carolina/Documents/Research/Sarafu/Exploration/scripts/SM'
```

In [2]:

```
import numpy as np
from collections import Counter
import pandas as pd
import json
import re
import os
import math
import random 
import networkx as nx
import datetime
import calendar
import matplotlib.pyplot as plt
import seaborn as sns
%matplotlib inline
```

In [3]:

```
submodules = [(1, 1), (1, 2), (1, 3), (1, 4), (1, 5), (1, 6), 
              (1, 7), (1, 8), (1, 9), (1, 10), (1, 13), 
              (2, 1), (2, 2), (2, 3), (2, 4), (2, 5), (2, 6), 
              (2, 7), (2, 8), (2, 9), (2, 10), (2, 11), 
              (2, 12), (2, 13), (2, 14), (2, 15), 
              (3, 1), (3, 2), (3, 3), (3, 4), (3, 6), 
              (4, 1), (4, 2), (4, 3), (4, 4), 
              (5, 1), (5, 2)]
```

In [19]:

```
# Define directories
homedir = '/Users/carolina/Documents/Research/Sarafu/Exploration/scripts/output/'
```

In [5]:

```
colors_dict = {'Mukuru Nairobi':"#85BD39",
         'Kinango Kwale':"#D390F8",
         'Kilifi/Nyanza/Misc Nairobi':"#303030",
         'Kisauni Mombasa':"#54C0F8",
         'Turkana':"#F28D25"}
```

In [6]:

```
regions = {1:'Mukuru Nairobi',
               2:'Kinango Kwale',
               3:'Kilifi/Nyanza/Misc Nairobi',
               4:'Kisauni Mombasa',
               5:'Turkana'}
```

---

In [20]:

```
def CycleCounts(model_type):
    
    text_file = model_type+'_cycles.txt'
    df = pd.DataFrame()
    
    with open(homedir+text_file,'r') as f:
        content = f.read()
        f.close()
        lines = content.split('\n')
        for l in range(len(lines[1:])):
            row = [i for i in lines[1:][l].split(',')[1:]]
            df = df.append(pd.Series(row), ignore_index=True)
        
    if model_type == 'empirical': 
        df.columns = ['cyc2', 'cyc3', 'cyc4', 'cyc5', 'n', 'm', 'mod_n', 
                      'submod_n']
    else:
        df.columns = ['cyc2', 'cyc3', 'cyc4', 'cyc5', 'n', 'm', 'mod_n', 
                      'submod_n', 'model_n']
        df['model_n'] = df['model_n'].apply(lambda x: int(x))
    
    for c in ['mod_n', 'submod_n', 'n', 'm']:
        df[c] = df[c].apply(lambda x: int(x))
    for c in ['cyc2', 'cyc3', 'cyc4', 'cyc5']:
        df[c] = df[c].apply(lambda x: float(x))
        
    return df
```

In [21]:

```
def Cycle_ZedScore(null_model_type):
    
    density_df = pd.DataFrame()
    for s in range(len(submodules)):
        module_n = submodules[s][0]
        submodule_n = submodules[s][1]
        emp = CycleCounts(model_type='empirical')
        emp = emp.loc[(emp.mod_n == module_n)&(emp.submod_n == submodule_n)]
        exp = CycleCounts(model_type=null_model_type)
        exp = exp.loc[(exp.mod_n == module_n)&(exp.submod_n == submodule_n)]
        
        for l in range(2,6):
            empirical = float(emp['cyc'+str(l)])
            expected = exp['cyc'+str(l)].apply(lambda x: float(x))
            
            try:
                density = (empirical - np.mean(expected)) / np.std(expected)
            except ZeroDivisionError:
                density = 'NaN'
                
            row = [empirical, np.mean(expected), np.std(expected), density, l, module_n, submodule_n]
            density_df = density_df.append(pd.Series(row), ignore_index=True)
            
    density_df.columns = ['emp', 'mean_exp', 'std_exp', 'zed', 'cyc_length', 'mod_n', 'submod_n']
    density_df['region'] = density_df.mod_n.apply(lambda x: regions[x])
    density_df['zed'] = density_df.zed.apply(lambda x: float(x))
        
    return density_df
```

In [ ]:

```
zed_scores_ER = Cycle_ZedScore(null_model_type='ER')
zed_scores_ERGs = Cycle_ZedScore(null_model_type='ERGs')
```

---

In [23]:

```
fig, axes = plt.subplots(nrows=1, ncols=1, figsize=(5, 5))
#
plotC = sns.stripplot(x="cyc_length", y="zed", 
              hue="region", data=zed_scores_ERGs, 
              palette=colors_dict);
plotC.set_ylabel('$\it{Z-}$score', fontsize=15)
plotC.legend(fontsize=12, frameon=False);
plotC.set_xlabel('Cycle Length', fontsize=14);
plotC.axhline(y=0, color='black', linestyle='--')
plotC.spines[['right', 'top']].set_visible(False)
#
plt.tight_layout()
# save
filepath = os.path.join(homedir,"correct_version_only_ERGs_only_zed.pdf")
plt.savefig(filepath,  bbox_inches='tight');
```

In [24]:

```
fig, axes = plt.subplots(nrows=1, ncols=2, figsize=(10, 5), sharey=False)
# 1
plotC = sns.stripplot(x="cyc_length", y="zed", 
              hue="region", data=zed_scores_ER.loc[zed_scores_ER.zed > 0], 
              palette=colors_dict, ax=axes[0]);
plotC.set_ylabel('$\it{Z-}$score', fontsize=15)
plotC.legend([],[], frameon=False);
plotC.set(yscale='log')
plotC.set_title('ER Null Model', fontsize=15)
plotC.set_xlabel('Cycle Length', fontsize=14);
plotC.axhline(y=0, color='black', linestyle='--')
plotC.spines[['right', 'top']].set_visible(False)
# 2
plotC = sns.stripplot(x="cyc_length", y="zed", 
              hue="region", data=zed_scores_ERGs, 
              palette=colors_dict, ax=axes[1]);
plotC.set_ylabel('$\it{Z-}$score', fontsize=15)
plotC.set(yscale='linear')
plotC.set_title('RD Null Model', fontsize=15)
plotC.legend(fontsize=12, frameon=False);
plotC.set_xlabel('Cycle Length', fontsize=14);
plotC.axhline(y=0, color='black', linestyle='--')
plotC.spines[['right', 'top']].set_visible(False)
#
plt.tight_layout()
# save
filepath = os.path.join(homedir,"correct_version_only_zed.pdf")
plt.savefig(filepath,  bbox_inches='tight');
```

In [ ]:

```

```

In [ ]:

```

```
