# Supplementary material for: Circulation of a digital community currency
Source: Sci Rep. 2023 Apr 11;13:5864. doi: 10.1038/s41598-023-33184-1 (PMC10088680; doi:10.1038/s41598-023-33184-1)
Supplement: Supplementary file 6 — Supplementary Information 6. [file 41598_2023_33184_MOESM6_ESM.pdf]

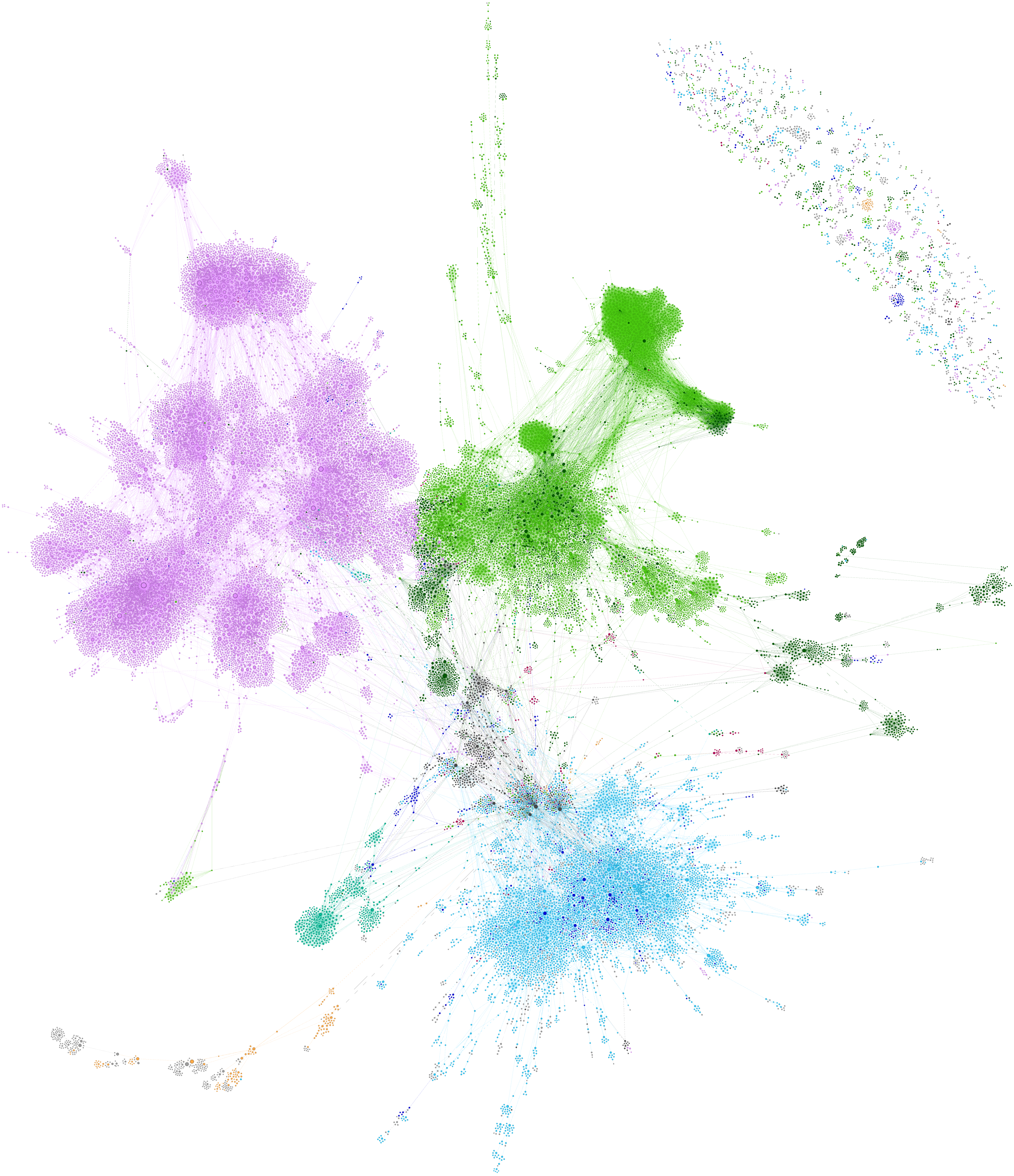

IN–DEGREE

```
========== Tail Index Estimation ==========
Number of data entries: 40657
========================
Selected AMSE border value: 1.0000
Selected fraction of order statistics boundary for AMSE
minimization: 0.2782
========================
Adjusted Hill estimated gamma: 2.908313858829886
**********
Moments estimated gamma: 2.884987476455898
**********
Kernel–type estimated gamma: 3.7329510844543194
**********
Elapsed time (total): 17.022118091583252
```

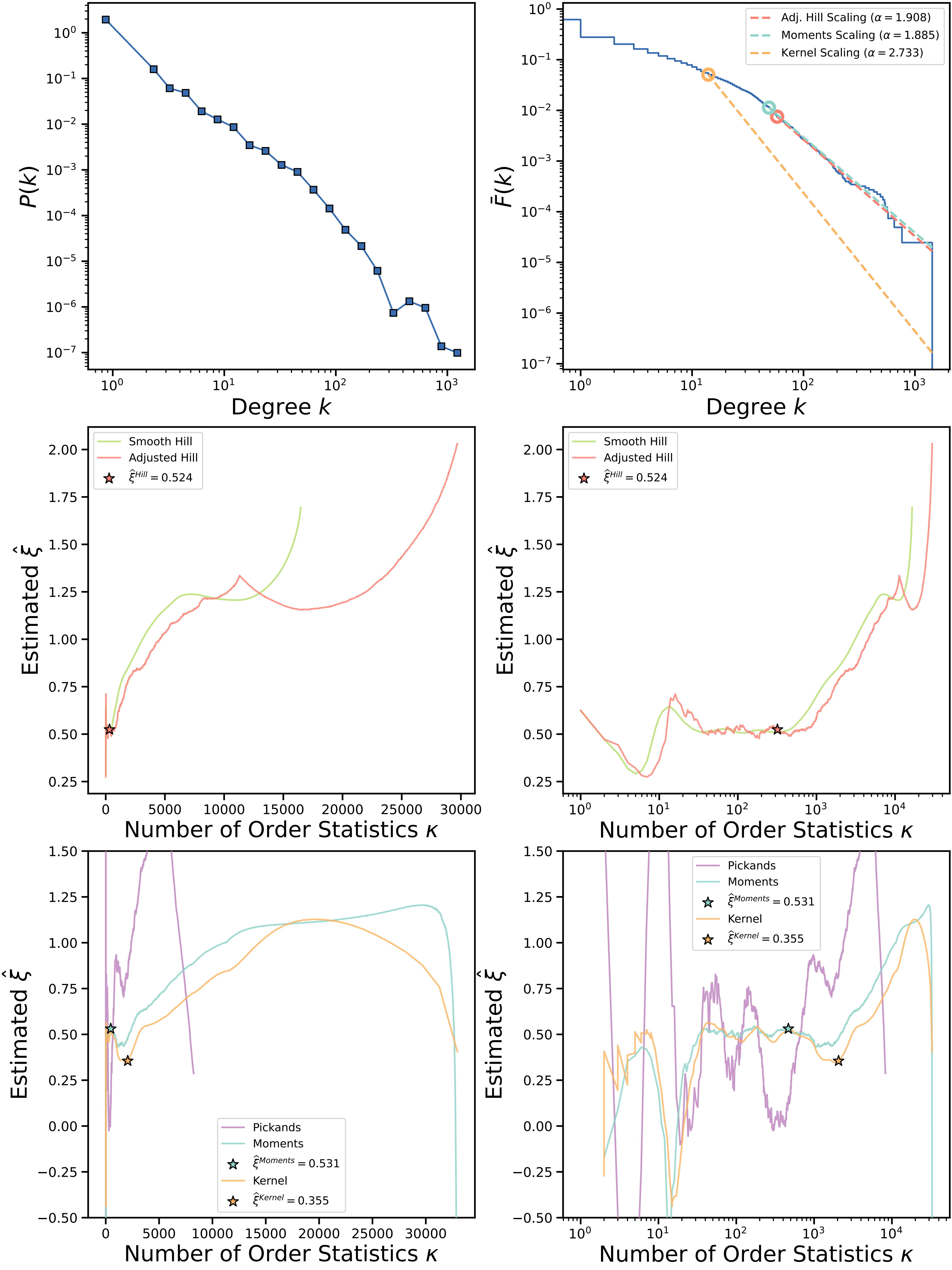

$P(k)$
Degree k
$\bar{F}(k)$
Adj. Hill Scaling ($\alpha = 1.908$)
Moments Scaling ($\alpha = 1.885$)
Kernel Scaling ($\alpha = 2.733$)
Smooth Hill
Adjusted Hill
$\hat{\xi}^{Hill} = 0.524$
Estimated $\hat{\xi}$
Number of Order Statistics κ
Pickands
Moments
$\hat{\xi}^{Moments} = 0.531$
Kernel
$\hat{\xi}^{Kernel} = 0.355$

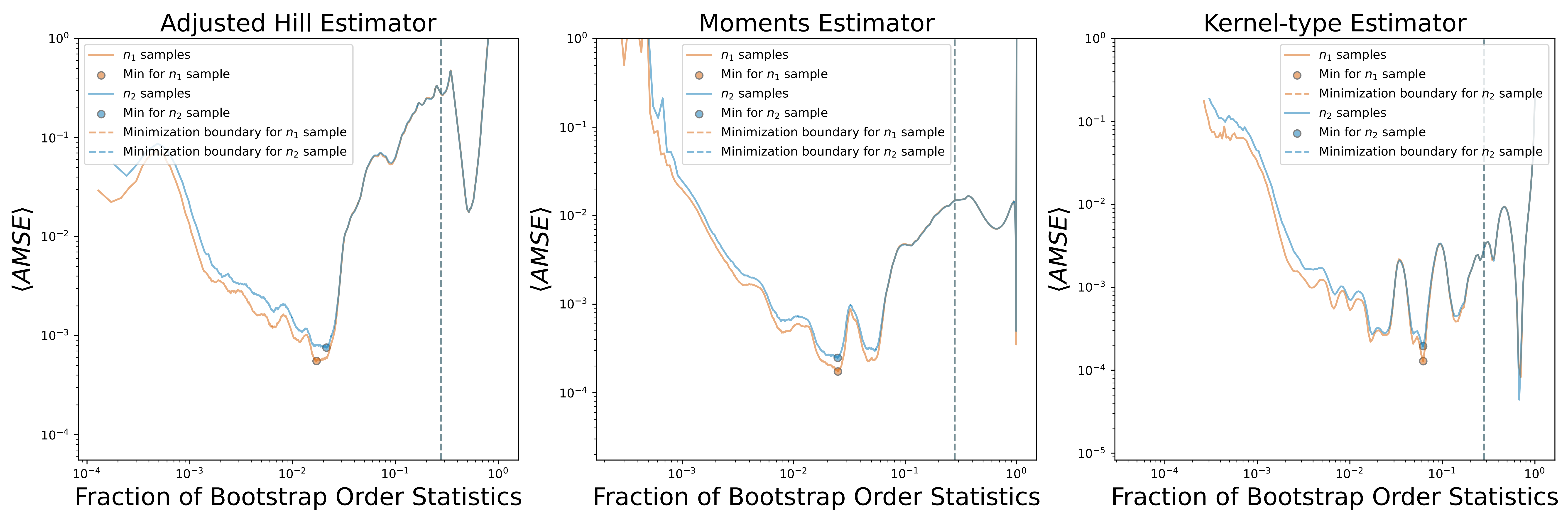

Adjusted Hill Estimator
Moments Estimator
Kernel-type Estimator
n_1 samples
Min for n_1 sample
n_2 samples
Min for n_2 sample
Minimization boundary for n_1 sample
Minimization boundary for n_2 sample
$\langle AMSE \rangle$
Fraction of Bootstrap Order Statistics

OUT-DEGREE

```
========== Tail Index Estimation ==========
Number of data entries: 40657
========================
Selected AMSE border value: 1.0000
Selected fraction of order statistics boundary for AMSE
minimization: 0.5139
========================
Adjusted Hill estimated gamma: 2.2191950309453596
**********
Moments estimated gamma: 2.9357202207088573
**********
Kernel-type estimated gamma: 2.2051767931594997
**********
Elapsed time (total): 19.610251903533936
```

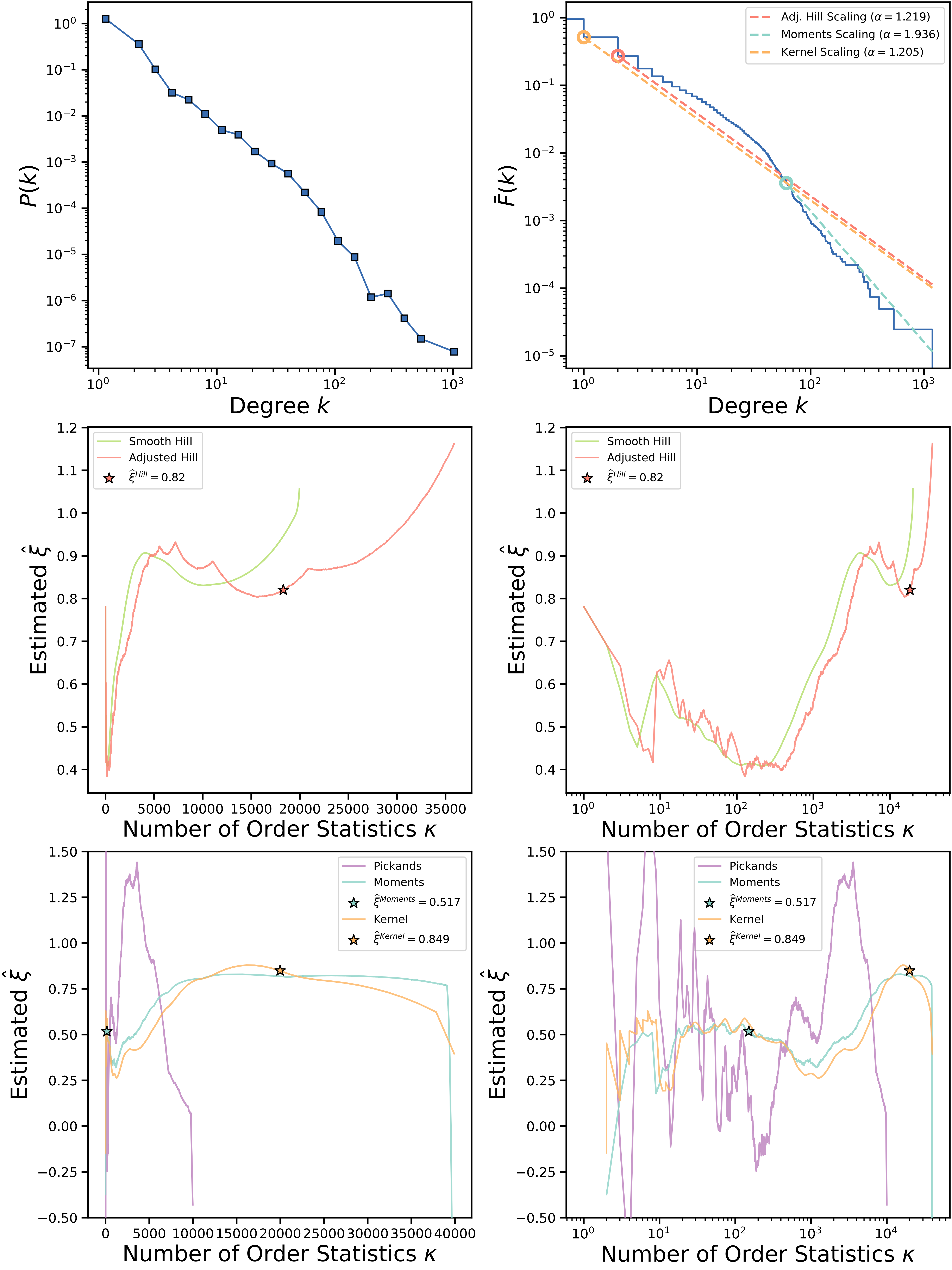

P(k)
Degree k
F̄(k)
Adj. Hill Scaling (α = 1.219)
Moments Scaling (α = 1.936)
Kernel Scaling (α = 1.205)
Smooth Hill
Adjusted Hill
ξ̂^Hill = 0.82
Estimated ξ̂
Number of Order Statistics κ
Pickands
Moments
ξ̂^Moments = 0.517
Kernel
ξ̂^Kernel = 0.849

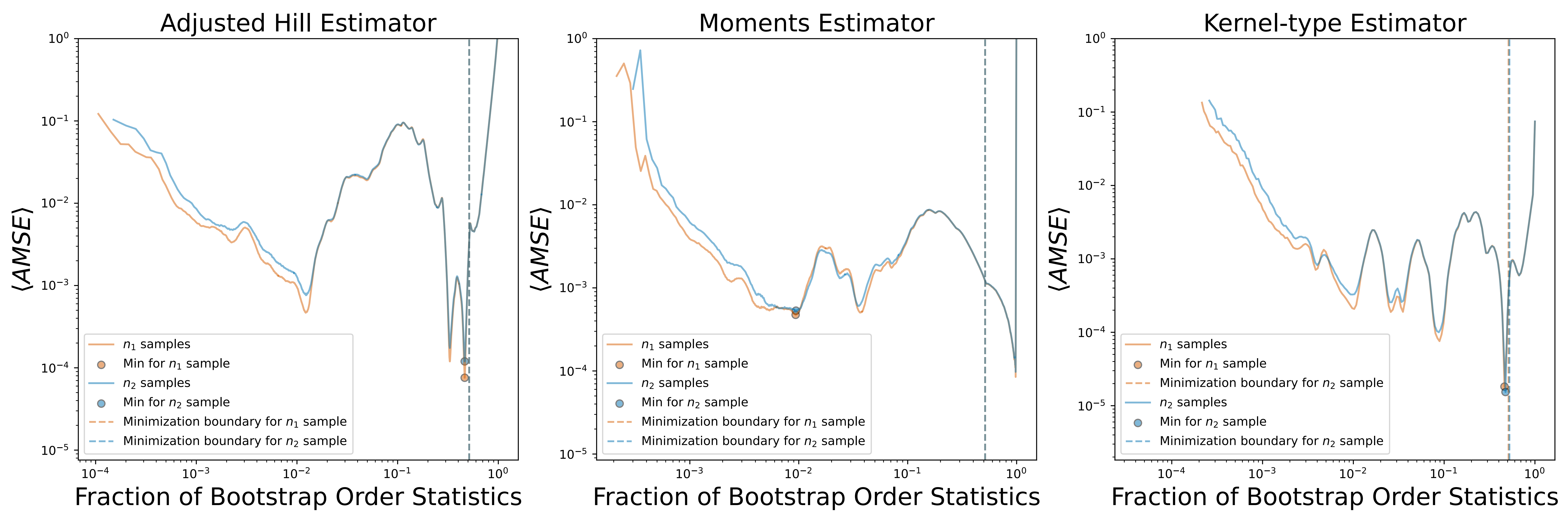

Adjusted Hill Estimator
Moments Estimator
Kernel-type Estimator
⟨AMSE⟩
Fraction of Bootstrap Order Statistics
n_1 samples
Min for n_1 sample
n_2 samples
Min for n_2 sample
Minimization boundary for n_1 sample
Minimization boundary for n_2 sample

IN-VOLUME

```
========== Tail Index Estimation ==========
Number of data entries: 40657
========================
Selected AMSE border value: 1.0000
Selected fraction of order statistics boundary for AMSE
minimization: 0.5892
========================
Adjusted Hill estimated gamma: 3.2612679819201866
**********
Moments estimated gamma: 4.713671043400444
**********
Kernel-type estimated gamma: 2.54980866958007
**********
Elapsed time (total): 17.036975860595703
```

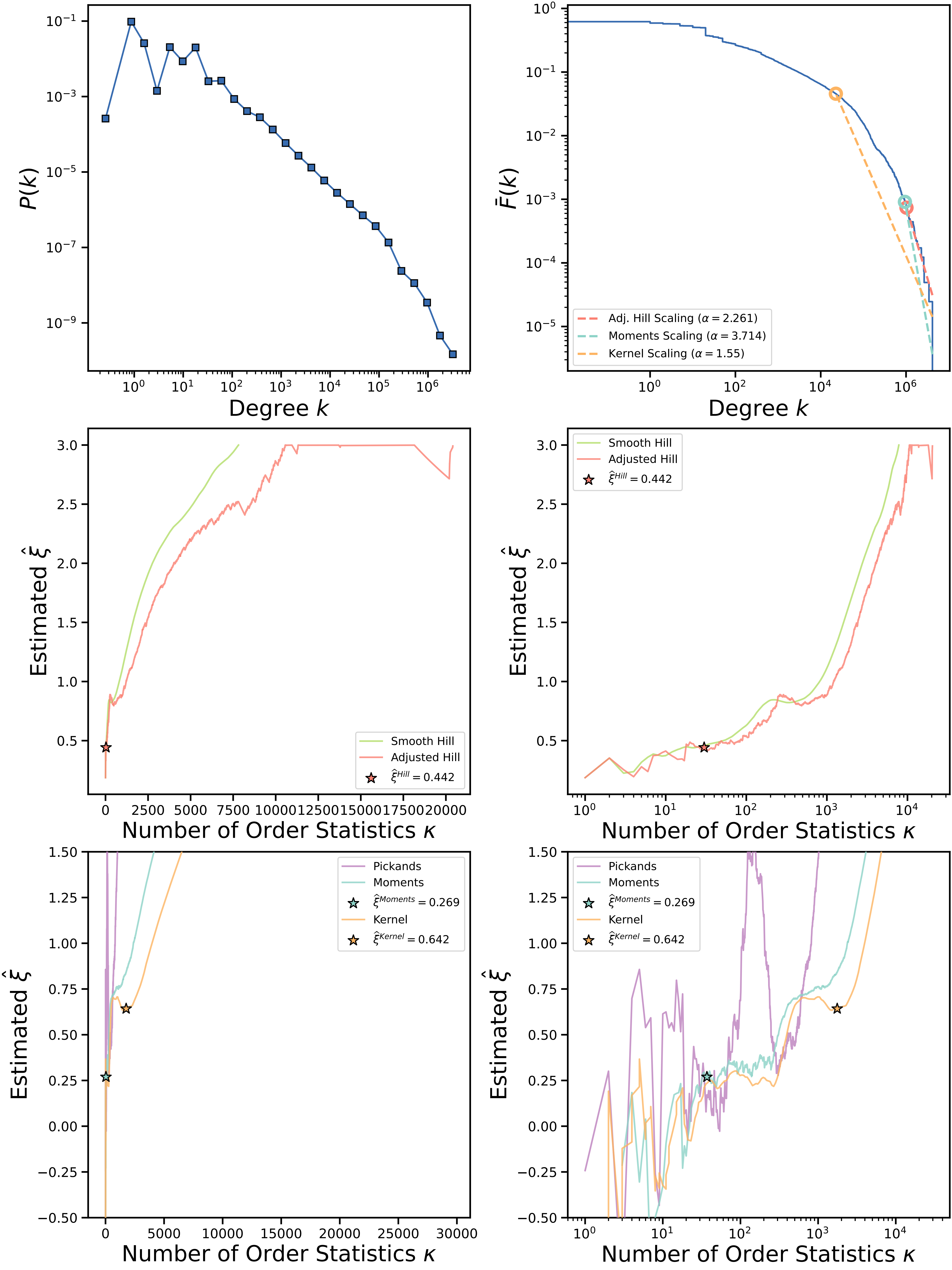

P(k)
Degree k
F̄(k)
Adj. Hill Scaling (α = 2.261)
Moments Scaling (α = 3.714)
Kernel Scaling (α = 1.55)
Estimated ξ̂
Number of Order Statistics κ
Smooth Hill
Adjusted Hill
ξ̂^Hill = 0.442
Pickands
Moments
ξ̂^Moments = 0.269
Kernel
ξ̂^Kernel = 0.642

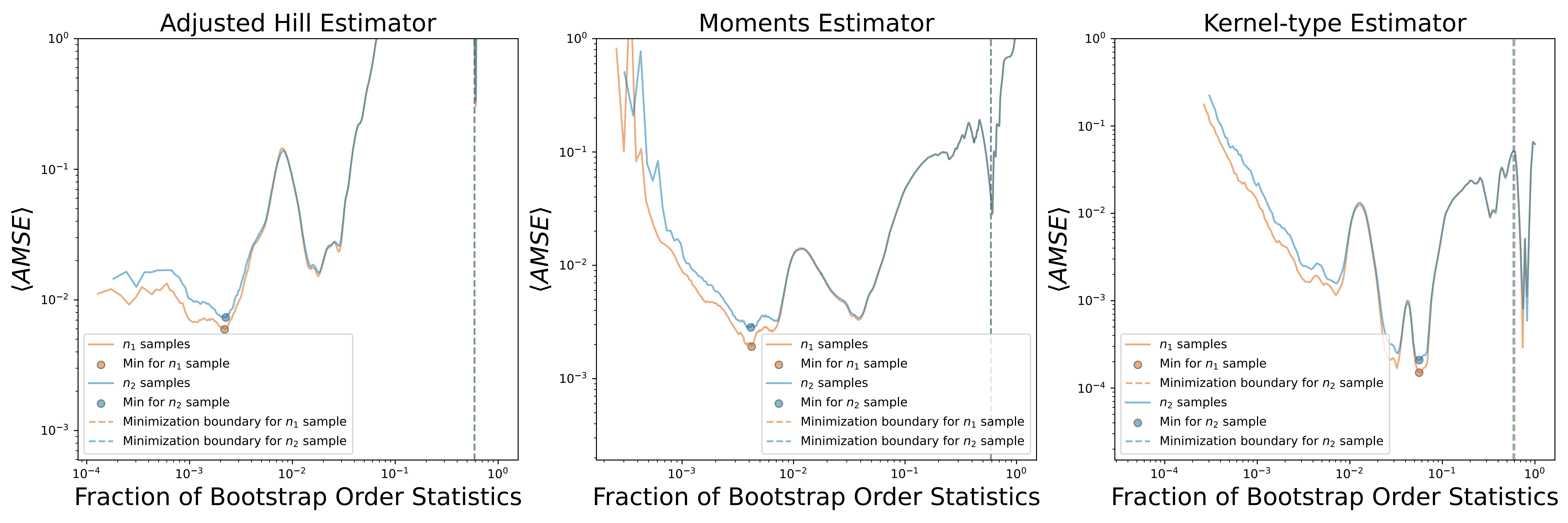
Adjusted Hill Estimator
Moments Estimator
Kernel-type Estimator
⟨AMSE⟩
Fraction of Bootstrap Order Statistics
n_1 samples
Min for n_1 sample
n_2 samples
Min for n_2 sample
Minimization boundary for n_1 sample
Minimization boundary for n_2 sample

```
OUT-VOLUME

========== Tail Index Estimation ==========
Number of data entries: 40657
========================
Selected AMSE border value: 1.0000
Selected fraction of order statistics boundary for AMSE
minimization: 0.5892
========================
Adjusted Hill estimated gamma: 3.3278662702825734
**********
Moments estimated gamma: 4.557046882046343
**********
Kernel-type estimated gamma: 2.5290322564723553
**********
Elapsed time (total): 17.559387922286987
```

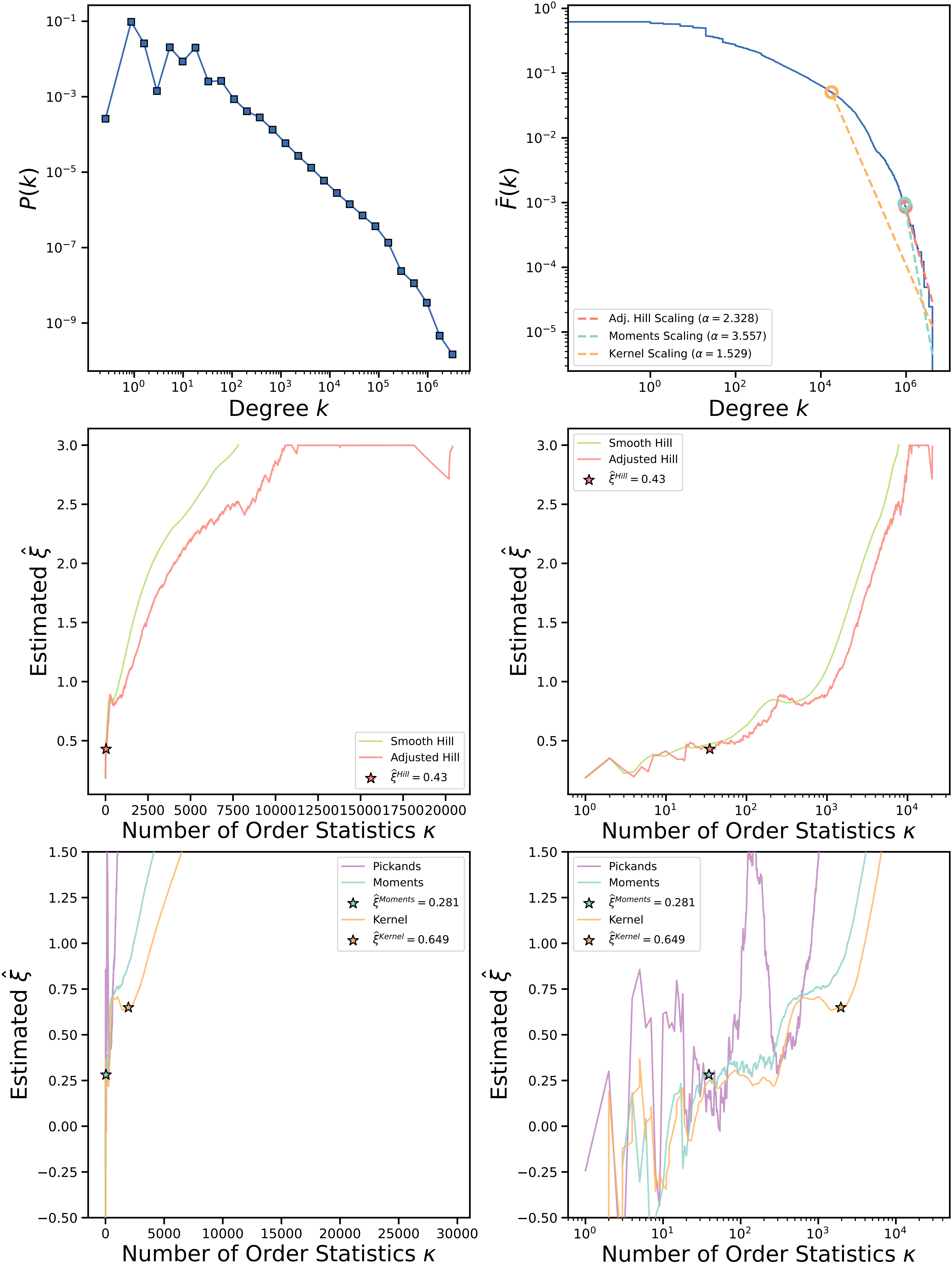

P(k)
Degree k
F̄(k)
Adj. Hill Scaling (α = 2.328)
Moments Scaling (α = 3.557)
Kernel Scaling (α = 1.529)
Estimated ξ̂
Number of Order Statistics κ
Smooth Hill
Adjusted Hill
ξ̂^Hill = 0.43
Pickands
Moments
ξ̂^Moments = 0.281
Kernel
ξ̂^Kernel = 0.649

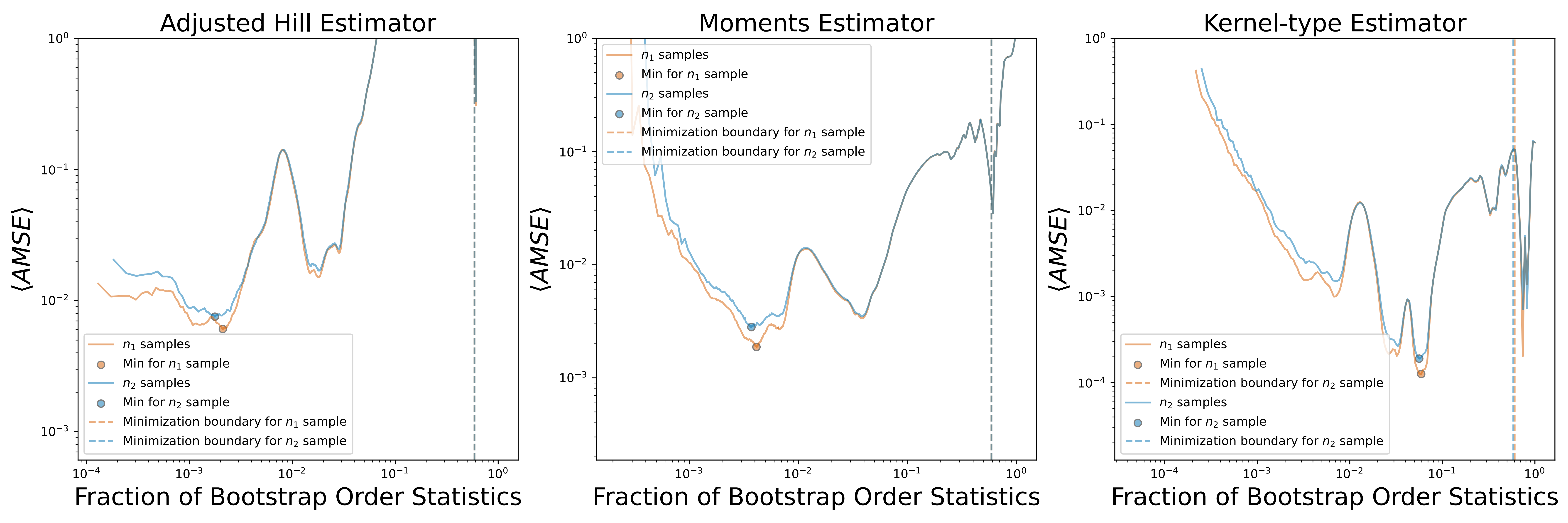

Adjusted Hill Estimator
Moments Estimator
Kernel-type Estimator
⟨AMSE⟩
Fraction of Bootstrap Order Statistics
n_1 samples
Min for n_1 sample
n_2 samples
Min for n_2 sample
Minimization boundary for n_1 sample
Minimization boundary for n_2 sample
Minimization boundary for n_2 sample
